# Electrospun eri silk fibroin scaffold coated with hydroxyapatite for bone tissue engineering applications

**DOI:** 10.1186/2194-0517-2-6

**Published:** 2013-03-08

**Authors:** Muthumanickkam Andiappan, Subramanian Sundaramoorthy, Niladrinath Panda, Gowri Meiyazhaban, Sofi Beaula Winfred, Ganesh Venkataraman, Pramanik Krishna

**Affiliations:** 1 Department of Textile Technology, Anna University, Chennai, 600025 India; 2 Department of Biotechnology and Medical Engineering, NIT, Rourkela, 769008 India; 3 Department of Human Genetics, Sri Ramachandra University, Chennai, 600116 India

**Keywords:** Cell attachment, Hemolysis, Hydroxyapatite scaffold, Swelling ratio, TGA

## Abstract

Natural biomaterials such as collagen, silk fibroin, and chitosan, and synthetic biopolymers such as polylactic acid, polycaprolactone, polyglycolic acid, and their copolymers are being used as scaffold for tissue engineering applications. In the present work, a fibrous mat was electrospun from eri silk fibroin (ESF). A composite of hydroxyapatite (Hap) and the ESF scaffold was prepared by soaking the ESF scaffold in a solution of calcium chloride and then in sodium diammonium phosphate. The average tensile stress of the pure ESF and hydroxyapatite-coated ESF scaffold (ESF-Hap) was found to be 1.84 and 0.378 MPa, respectively. Pure ESF and ESF-Hap scaffolds were evaluated for their characteristics by a themogravimetric analyzer and Fourier transform infrared spectroscope. The crystallinity and thermal stability of the ESF-Hap scaffold were found to be more than that of uncoated eri silk nanofiber scaffold. The water uptake of the pure ESF and ESF-Hap scaffolds was found to be 69% and 340%, respectively, in distilled water as well as phosphate buffer saline. The hemolysis percentage of both scaffolds was less than 5%, which indicate their good blood compatibility. The cytocompatibility studied by 3-(4,5-dimethyl) thiazol-2-yl-2,5-dimethyl tetrazolium bromide assay showed that the scaffold is biocompatible. To assess cell attachment and growth on the scaffold, human mesenchymal stem cells were cultured on the scaffolds. The results from scanning electron microscopy and fluorescent microscopy showed a notable cellular growth and favorable morphological features. Hence, the ESF-Hap scaffold is better suited for cell growth than the pure ESF scaffold.

**Electronic supplementary material:**

The online version of this article (doi:10.1186/2194-0517-2-6) contains supplementary material, which is available to authorized users.

## Background

Biomaterials that are aimed at providing replacement for tissues and organs damaged or lost as a consequence of disease, aging, or accident, play a more and more important role in tissue engineering (Du et al. 2009). Natural bone is a complex inorganic and organic nanocomposite material, in which about 70 wt.% of hydroxyapatite (Hap) nanocrystals and about 30 wt.% of collagen fibrils are well organized into a hierarchical architecture over several length scales (Du et al. 2000; Fan et al. 2010). In recent years, tissue engineering has revolutionized the direction of research for orthopedic applications because of the success of nanotechnological advancements in creating fabrication techniques for nanoscale materials, such as nanofibrous scaffolds by electrospinning (Wei et al. 2011; Woo et al. 2003). Bioactive ceramics, such as Hap and bioglass, are widely used as bone substitute materials as they can bond directly to living bone (Marcolongo et al. 1997). Hydroxyapatite possesses higher mechanical strength and better stability, which is used for tissue engineering during the last two decades. Synthetic polymers such as polylactic acid (PLA), poly(*e*-caprolactone) (PCL), and poly(lactide-*co*-glycolide) have good mechanical and biocompatibility properties; however, they lack hydrophilic properties and cell affinity (Venugopal et al. 2005). Silk fibroin is a typical fibrous protein that has recently been studied as a scaffold for tissue engineering because of its excellent biocompatibility, bio-absorbability, and low level of inflammatory potential (Altman et al. 2003; Wei et al. 2011; Meinel et al. 2005; Panilaitis et al. 2003). Regenerated silk fibroin obtained from mulberry (*Bombyx mori*) silk is the most extensively characterized silk fibroin with outstanding biocompatible properties, and its composite with hydroxyapatite has a high osteoconductivity (Weng et al. 1997; Furuzono et al. 2000; Ren et al. 2007). In the earlier studies, regenerated mulberry silk fibroin and *Antheraea pernyi* silk fibroin were used to synthesize hydroxyapatite-mineralized fibroin for bone tissue engineering. The wild silks such as tasar, eri, and muga have been used as a textile material for a long period. The major amino acid (glycine, alanine, and serine) composition of eri silk (84.26%) is higher than that of mulberry (82.8%), muga silk (67.77%), and tasar silk (72.06%). The moisture recovery of the eri silk fibroin is higher than that of mulberry silk and muga silk (Sen and Murugesh Babu 2000ilk fibroin (Li et al.). The tripeptide sequence of arginine, glycine, and aspartic acid is higher in mulberry *s*^2008^). The study on the comparison of mulberry silk with eri silk showed that cell attachment, binding, and spreading of L6 fibroblast cells on the eri silk scaffold were better than those on the mulberry silk fibroin, and cell viability was found to be better on eri silk fibroin scaffold (Muthumanickkam et al.2012a,b). The aim of the present study is to (1) develop hydroxyapatite-coated eri silk fibroin (ESF-Hap) scaffold by alternate soaking of ESF scaffold in CaCl_2_ and Na_2_HPO_4_ and (2) to assess the physical and chemical properties, blood compatibility, cell attachment, and cell viability of the pure ESF and ESF-Hap scaffolds.

## Methods

### Preparation of the eri silk nanofiber scaffold

The eri silk was degummed with sodium carbonate solution boiling at 75°C for 30 min to remove the sericin from the silk filament. The degummed solution was maintained at a pH level of 8.5 to 9.0. Then the degummed silk (silk fibroin) was dissolved in trifluoroacetic acid (99.5%). For electrospinning, the fibroin solution was aspirated with a 2-ml syringe having a diameter of 0.55 mm. The syringe was fixed on the infusion pump in a vertical position. Initially, spraying of solution and formation of beads occurred while electrospinning from the silk fibroin solution. A number of trails have been conducted to optimize the concentration of fibroin in the solution and electrospinning process parameters such as distance between the syringe and collection plate, voltage, and flow rate such that the fibers were formed in the nanoscale without the formation of beads. The optimum concentration of polymer in trifluoroacetic acid was found to be 13% (wt/vol.). The distance between the syringe and the collecting drum was kept at 15 cm, and a 20-kV supply was applied between the syringe and the collecting drum (Muthumanickkam et al. 2010). The flow rate of the solution was maintained at 1.0 ml/h. The scaffold obtained had a thickness of 0.25 mm ± 0.01 mm. The porosity of the scaffold was calculated using Equation 1:

1 $$\mathrm{Porosity}\;\left(\%\right)=\left[1-\left(\mathrm{Apparent}\;\mathrm{density}\;/\mathrm{Bulk}\;\mathrm{density}\;\mathrm{of}\;\mathrm{fiber}\right)\right]\times 100$$

The apparent density is the ratio of the mass to the volume of the scaffold. The bulk density of the silk fibroin is 1.25 g/cm^3^.

### Preparation of eri silk fibroin and hydroxyapatite composite

The electrospun eri silk fibroin scaffold was immersed in 0.5 M of calcium chloride solution in a Tris buffer for 12 h at a pH of 10.4. The scaffold was rinsed with distilled water and subsequently immersed in 0.5M of Na_2_HPO_4_ solution in a Tris buffer for 12 h at a pH of 10.4. The scaffold was again rinsed with distilled water. The above mentioned steps were repeated three times.

### Characterization of scaffolds for physical and chemical properties

The pure eri silk fibroin and hydroxyapatite composite scaffolds were studied for their functional properties using a Fourier transform infrared spectroscope (FTIR) (PE 1600, Perkin Elmer, Waltham, MA, USA). The wavelength was ranged between 4,000 and 400 cm^-1^. The thermal stability of the scaffolds was analyzed using a themogravimetric analyzer (Q500 thermal analyzer, TA Instruments, New Castle, DE, USA). The study on thermal stability of the scaffold was carried out at a heating rate of 20°C/min. The ESF and ESF-Hap scaffolds were evaluated using an X-ray diffractometer (D8, Bruker, Madison, WI, USA) with CuKα radiation (λ = 1.54 Å). The scanning was carried out at a speed of 0.04°/s with a measurement range of 1° to 70°. The crystal size was measured using Equation 2.

2 $$\mathrm{Crystal}\;\mathrm{size}\;Å=\frac{\mathrm{K\lambda}}{\beta \;\cos\theta }$$

where *λ*, *β*, and *θ* are the wavelength of CuKα, X-ray diffraction broadening, and observed peak angle, respectively. The crystal size was measured from the first three peaks of the respective X-ray diffractogram. The full width at half maximum was determined using Fityk software.

The scaffold was tested for tensile properties at standard atmospheric conditions, using an Instron 3369 (Norwood, MA, USA) tensile strength tester. The scaffold was cut into the specimen size of 10 × 50 mm. Glue tapes were fixed at the top and bottom of the scaffold, where it was clamped on the jaw of the tester. The gauge length was maintained at 30 mm, and the test speed was kept at 20 mm/min. Water uptake is an indicator of the hydrophilic characteristic of the material, which is essential for applications such as tissue engineering and wound dressings. The scaffold was immersed in (1) phosphate buffered saline (PBS) maintained at pH 7.4 and 37°C, and (2) distilled water maintained at 37°C for different immersion periods of 1, 2, 3, 4, 5, 6, 7, 8, 24, 48, and 96 h. The water uptake of pure ESF and ESF-Hap scaffolds was measured until the material reached the saturated condition. The wet weight (*W*_T_), after the removal of excess amount of water using a filter paper, and the dry weight of the scaffold (*W*_D_) were measured. The water uptake percentage for different immersion periods with respect to bone dry weight was determined using Equation 3.

3 $$\mathrm{Water}\;\mathrm{uptake}\;\%\;=\;\frac{W_{T}\;-\;W_{D}}{W_{D}}\;\times \;100$$

### Bioactivity test of scaffolds

The pure ESF and ESF-Hap scaffolds were immersed in a 1.5 simulated body fluid solution (SBF) and kept at 37°C for 15 days. Then the scaffolds were removed from the SBF solution, rinsed with distilled water, and then air dried at ambient temperature. The scanning electron microscope (SEM) image was taken to visualize the deposition of the apatite layer on the scaffold.

### Hemolysis assay

Blood compatibility is one of the most important properties of biomedical materials. Hemolysis is the rupture of erythrocytes and release of their contents into surrounding fluid. The pure ESF and ESF-Hap scaffolds were subjected to a hemolysis assay, as per ISO 10993–4, to assess their blood compatibility. Human blood collected from a healthy volunteer and placed in a 3.8% sodium citrate-coated tube was diluted with PBS (pH 7.4) at a ratio of 1:20 (*v*/*v*). The blood diluted with PBS was taken as negative control, and the blood with Triton X was taken as positive control. The autoclaved pure ESF and ESF-Hap scaffolds were immersed in 100 μl of blood, and PBS solution followed by incubation at 37°C for 60 min. Then, the samples were spun at 3,000 rpm for 10 min. The optical density value (OD) of the supernatant was measured using a spectrophotometer at 545 nm, and the hemolysis percentage was estimated using Equation 4.

4 $$\mathrm{Hemolysis}\;\%=\frac{\mathrm{OD}\;\mathrm{value}\;\mathrm{of}\;\mathrm{sample}\;-\mathrm{OD}\;\mathrm{value}\;\mathrm{of}\;\mathrm{negative}}{\;\mathrm{OD}\;\mathrm{value}\;\mathrm{of}\;\mathrm{positive}-\mathrm{OD}\;\mathrm{value}\;\mathrm{of}\;\mathrm{negative}}\;\times \;100$$

### Platelet adhesion

Platelet adhesion is one of the important tests to evaluate the hemocompatibility of materials. Five milliliter of fresh human blood was collected from a healthy volunteer. The fresh blood was treated with 3.8% sodium citrate and spun at 3,000 rpm for 10 min at 4°C to obtain platelet-rich plasma, and then, it was placed on the scaffolds. Platelet-attached pure ESF and ESF-Hap scaffolds were washed twice with PBS and then immersed in PBS containing 2.5% glutaraldehyde (pH 7.4) overnight. They were subsequently dehydrated in gradient ethanol (20%, 40%, 60%, 80%, and 100%) for 15 min and then dried in vacuum. The morphology of the platelets that adhered on the scaffolds was characterized by the SEM analysis.

### hMSC attachment on the scaffolds

Cell culture-grade reagents (Sigma Aldrich, St. Louis, MO, USA) were used throughout this study. Human mesenchymal stem cells (hMSCs) were maintained in culture using Dulbecco's modified Eagle's medium (DMEM) supplemented with 10% fetal bovine serum (FBS), 2 mM of l-glutamine, 10,000 IU of penicillin, 10,000 μg/ml of streptomycin, and 25 μg/ml of amphotericin-B. Cylindrical disks of 6-mm diameter were cut from the electrospun fibrous mat with a dermal biopsy punch. Approximately 3.5 × 10^5^ cells/ml were seeded per disk placed in each well. After the desired number of days, the disks were removed from the culture media, washed twice with 1× PBS, and then fixed with 3% glutaraldehyde solution (diluted from 50% glutaraldehyde solution (Electron Microscopy Science, Hatfield, PA, USA) with PBS). Then the disks were subjected to gradient ethanol (20%, 50%, 70%, 90%, and 100%) for 10 min and refrigerated overnight at 4°C. The scaffolds were then coated with palladium and studied by SEM on the third, fifth, and seventh days.

### Fluorescence image of cell seeded in the scaffolds

The morphology of hMSCs cultured for different periods of time on ESF and ESF-Hap scaffolds was observed. Mesenchymal stem cells were seeded at a density of 5 × 10^4^ cells/disk (diameter, 6 mm) and cultured in DMEM + 10% FBS at 37°C for 3, 5, and 7 days. The unattached cells were removed by washing with PBS, and the attached cells were fixed with 4% formaldehyde in PBS. Cells were treated with 0.2% TritonX-100 (Sigma, T9284) in PBS, blocked in 2% denatured bovine serum albumin (dBSA), and probed for actin with Alexa 488-phalloidin (1:200; A12379, Invitrogen, Grand Island, NY, USA) in a 25-mM Tris buffer containing 2% dBSA for 45 min at 37°C. Nuclei were labeled with 20 μg/ml of DAPI (Invitrogen, D21490) in PBS for 4 min at room temperature followed by rinsing in the Tris buffer. Fluorescent images were taken with a CCD camera attached to a fluorescent microscope (Carl Zeiss, Oberkochen, Germany). The live cells were shown as fluorescent green, and the dead cells as red.

### MTT assay for scaffolds

In order to confirm cell compatibility of the ESF and ESF-Hap composite scaffolds, hMSCs were cultured on the scaffolds. The ESF and ESF-Hap scaffolds were soaked in ethanol and sterilized using ultraviolet light, followed by washing with sterile PBS(pH 7.4). The scaffold disk was placed in a 96-well tissue culture plate, followed by adding 100 μl of hMSC suspension with a concentration of about 5 × 10^5^ cells/ml in each well. The cells were incubated at 37°C with 5% CO_2_ for a period of 3, 5, and 7 days, respectively. The viability was measured using the 3-(4,5-dimethyl) thiazol-2-yl-2,5-dimethyl tetrazolium bromide (MTT, 5 mg/ml) method. The OD was measured at the wavelength of 595 nm using a spectrophotometric microplate reader (Perkin Elmer, Model: 2030 Explorer). The absorbance was proportional to the number of cells on the scaffolds. All samples were incubated at 37°C and 5% CO_2_. Three parallel experiments were carried out for each sample.

## Results and discussion

### Analysis of physical and chemical characteristics of pure ESF and ESF-Hap scaffolds

#### Surface characteristics of the scaffolds

**Figure 1:**
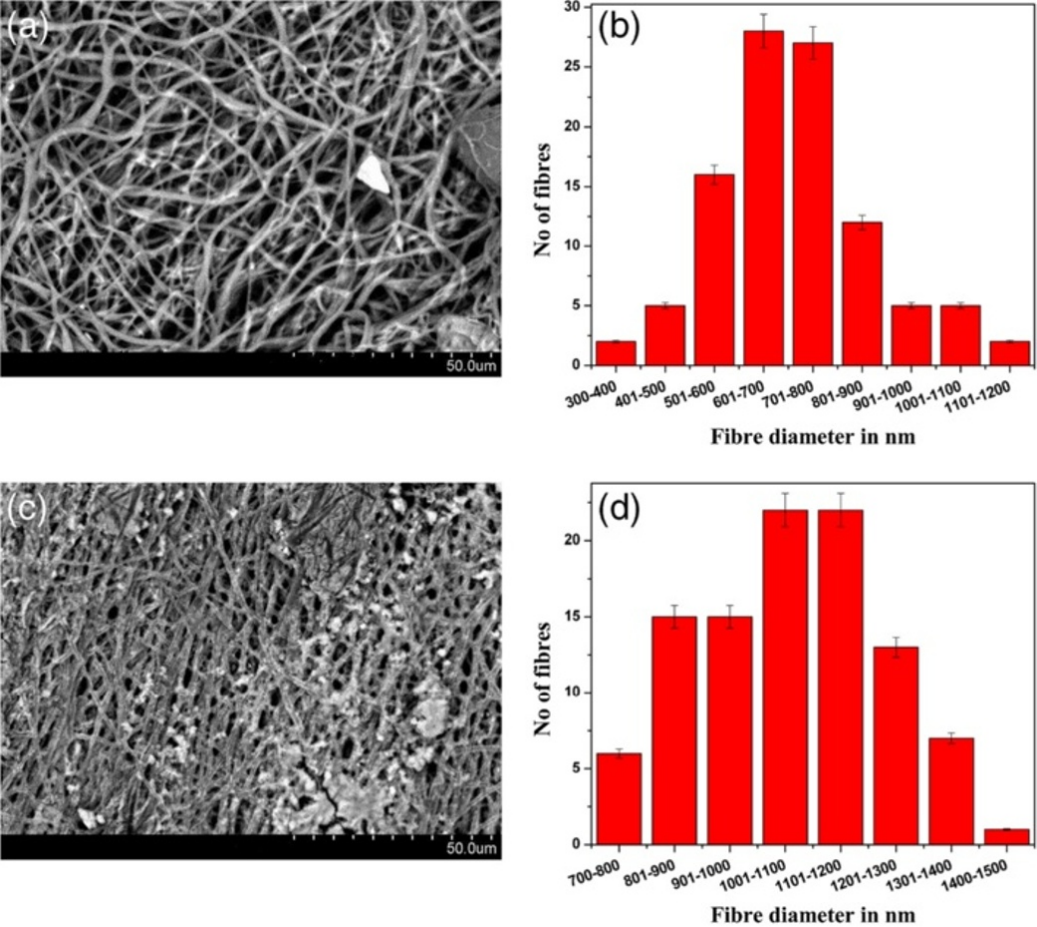
**Surface characteristics of the ESF and ESF-Hap scaffold.** (**a**) SEM image and (**b**) fiber diameter distribution of the pure ESF scaffold. (**c**) SEM image and (**d**) fiber diameter distribution of the ESF-Hap scaffold.

The SEM image and fiber diameter histogram of pure ESF are shown in Figure 1a,b, respectively. Figure 1c,d shows the SEM image and fiber diameter histogram of ESF-HaP scaffold. The Hap deposition on the fibers of the scaffold can be seen in Figure 1c. The histogram was constructed from 100 measures of diameter taken randomly from the SEM image. In the case of pure ESF, majority of the fibers have diameter in the range of 600 to 800 nm, and it is 1,000 to 1,200 nm for the ESF-Hap scaffold. The average fiber diameter of the ESF-Hap scaffold is higher than that of the pure ESF scaffold due to the presence of hydroxyapatite on the scaffold surface. The porosity of the scaffold was found to be 74% to 78%. Whang et al. (1999) found that 73.9% porosity was suitable for bone tissue engineering applications (Muthumanickkam et al.2012a,b).

#### Functional properties

**Figure 2:**
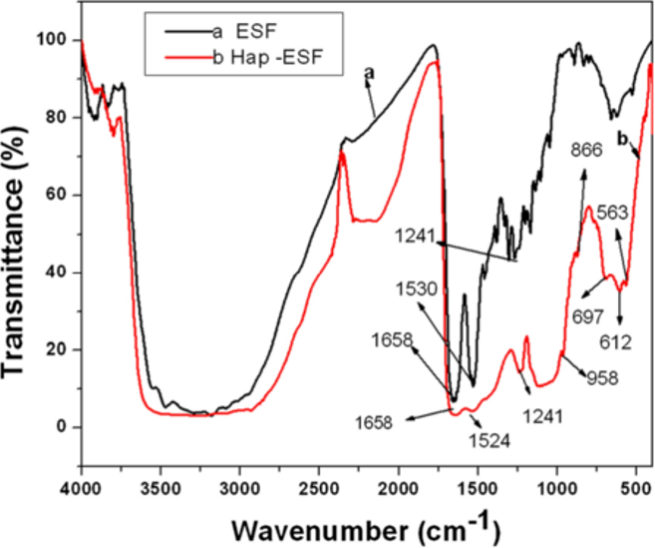
**FTIR spectra of pure ESF and ESF-Hap scaffold.**

The FTIR spectra of the pure ESF and ESF-Hap scaffolds are shown in Figure 2. The figure shows amide I absorption at 1,658 cm^-1^ (C=O stretch), amide II adsorption at 1,530 cm^-1^ (N-H bending), and amide III adsorption at 1,241 cm^-1^ (C-N stretching) for ESF scaffold. In the case of the ESF-Hap scaffold, amide I and amide III absorption happens at the same wave numbers as that of pure ESF scaffold, and amide II absorption is at 1,524 cm^-1^. However, the peak intensity of the ESF-Hap scaffold is less due to the presence of Hap in the ESF-Hap scaffold. The FTIR also shows that the stretch bands pertaining to trifluoroacetic acid (1,100 to 1,200 cm^-1^) used for preparing the polymer solution, which may be allergenic to be used as biomaterial, are not found in the electrospun fibrous scaffolds (Muthumanickkam et al. 2010). The band absorption in spectrum b (Figure 2) at 697, 612, and 563 cm^-1^ is due to O-P-O bending in the ESF-Hap scaffold. The band absorption at 959 cm^-1^ in spectrum b (Figure 2) corresponds to P-O stretching vibration due to the presence of PO_4_^3-^ group in the ESF-Hap scaffold. The C=O stretch vibration of amide I is shown as absorption at 1,658 cm^-1^, and the N-H bending of amide II is shown as absorption at 1,524 to 1,530 cm^-1^ in spectra a and b (Figure 2), respectively, for the pure ESF and ESF-Hap scaffolds. The intensity of the amide peaks of the ESF-Hap scaffold is less compared to that of the pure ESF, which is due to the formation of the bond between the Ca^+2^ ions and C=O (Wei et al. 2011; Ren et al. 2007). The oxygen atoms of the carboxyl and carbonyl groups present on the surface of the fibroin bind with the calcium ions, and they serve as the nucleation sites for apatite formation. Consequently, the Hap crystals precipitate on the surface the silk fibroin.

#### Thermal stability

**Figure 3:**
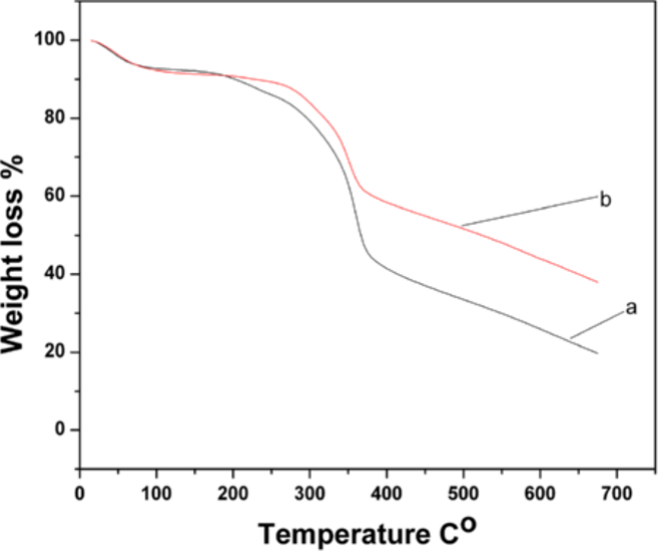
**Thermograms of pure ESF (curve a) and ESF-Hap scaffolds (curve b).**

The thermograms of pure ESF and ESF-Hap scaffolds are shown in Figure 3a,b, respectively. The figure shows that the weight loss is about 7% to 10% at 100°C for both ESF and ESF-Hap scaffolds due to the evaporation of water. The difference in weight loss between the pure ESF and ESF-Hap scaffolds is not significant at 100°C. The pure ESF scaffold starts to decompose at 250°C to 300°C, and its weight loss is about 20% from the original. On increasing the temperature, the pure ESF scaffold severely decomposes at 300°C to 350°C with a weight loss of about 55% from the original due to the breaking of the peptide bonds. The ESF-Hap scaffolds start to decompose at 300°C, and their weight loss is about 15% from the original. On increasing the temperature further, the ESF-Hap scaffold suffers a sharp decomposition at 375°C with a weight loss of about 40%. It is inferred that the thermal stability of the ESF-Hap scaffold is better than that of the pure ESF scaffold due to the presence of the inorganic salt of hydroxyapatite. This is similar to the findings of Wei (2011), who carried out the study on mulberry silk.

#### Structure

**Figure 4:**
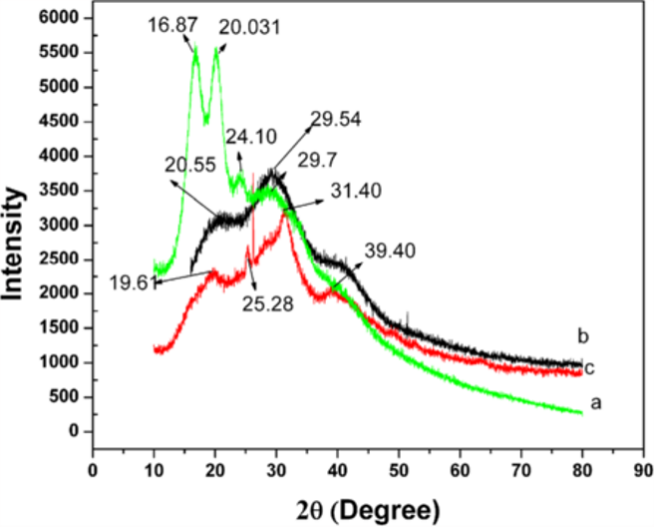
**X-ray diffraction.** Raw eri silk fibroin (curve a), ESF-Hap scaffold (curve b), and pure ESF scaffold (curve c).

Figure 4 (curves a, b, and c) shows the X-ray diffractogram of raw eri silk fibroin, ESF-Hap scaffold, and pure ESF scaffold. The diffractogram in Figure 4 (curve a) shows peaks (2*θ*) at 16.87° and 20.031°, and the corresponding space (*d*) at 5.30 and 4.50 Å respectively, indicating an α-helix structure. The peaks (2*θ*) at 24.10° and 29.7° and their corresponding space (*d*) at 3.771 and 3.13 Å indicate a β-sheet structure. Figure 4 (curve b) shows a peak at 20.55° (2*θ*) and the corresponding space (*d*) at 4.387 Å for the α-helix structure and diffraction at 29.54 (2*θ*) and space (*d*) at 12.3 Å for the β structure. Figure 4 (curve c) shows the diffraction peak for ESF-Hap at 19.3° (2*θ*) and its corresponding space(*d*) at 4.57 Å for the α helical structure, and three peaks at 25.28°, 31.40°, and 39.1° (2*θ*) are due to the presence of hydroxyapatite in the scaffolds (Wei et al. 2011; Ren et al. 2007; Zhao et al. 2009). From Equation 2, the crystal size of the ESF-Hap and pure ESF scaffolds were calculated, and the values are 57 and 25 Å, respectively. The crystal size of the ESF-Hap scaffold is higher due to the deposition of hydroxyapatite on the scaffold.

#### Tensile behavior

**Figure 5:**
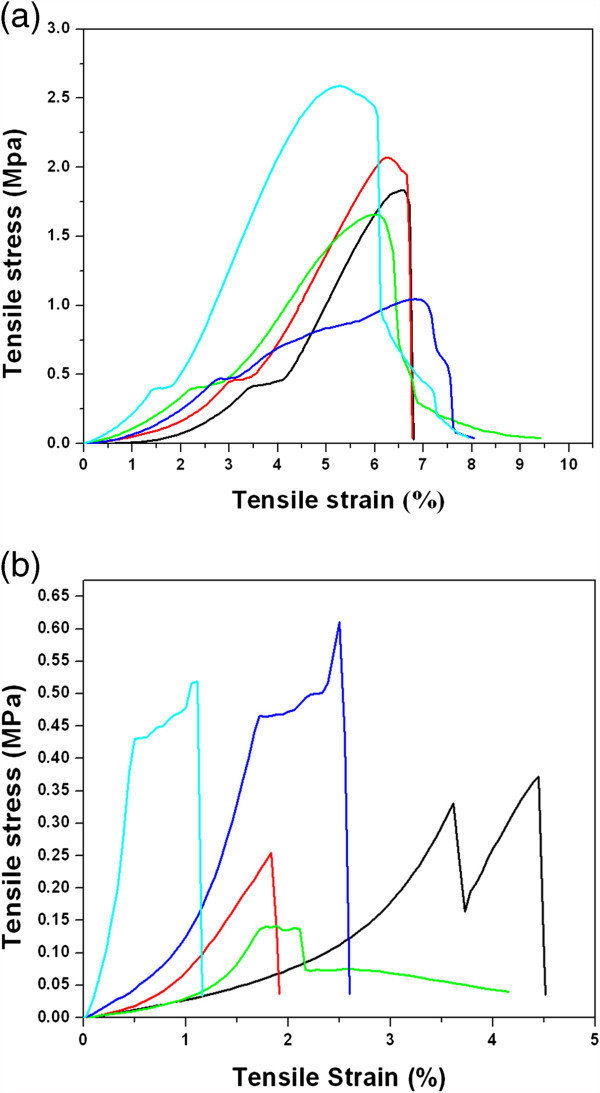
**Tensile behavior.** Stress–strain curve for (**a**) pure ESF and (**b**) ESF-Hap scaffolds. The individual lines in the diagram show the stress–strain behavior of individual test of samples.

Figure 5a,b shows the stress–strain diagram of the pure ESF and ESF-Hap scaffolds, respectively. The individual lines in the diagram show the stress–strain behavior of individual test of samples. The average tensile stress and tensile strain of the pure ESF and ESF-Hap scaffolds are 7.8% and 1.84 MPa, and 2.9% and 0.378 MPa, respectively. The pure ESF scaffold has higher tensile stress and strain than the ESF-Hap scaffold.

The decrease in strength and extensibility of the ESF-Hap scaffold is due to the increase in stiffness of the scaffold due to Hap coating. A similar trend was observed for electrospun PCL/Hap and Hap-treated electrospun PLA scaffolds (Zhao et al. 2009; Venugopal et al. 2008; Prabhakaran et al. 2009).

#### Water uptake

**Figure 6:**
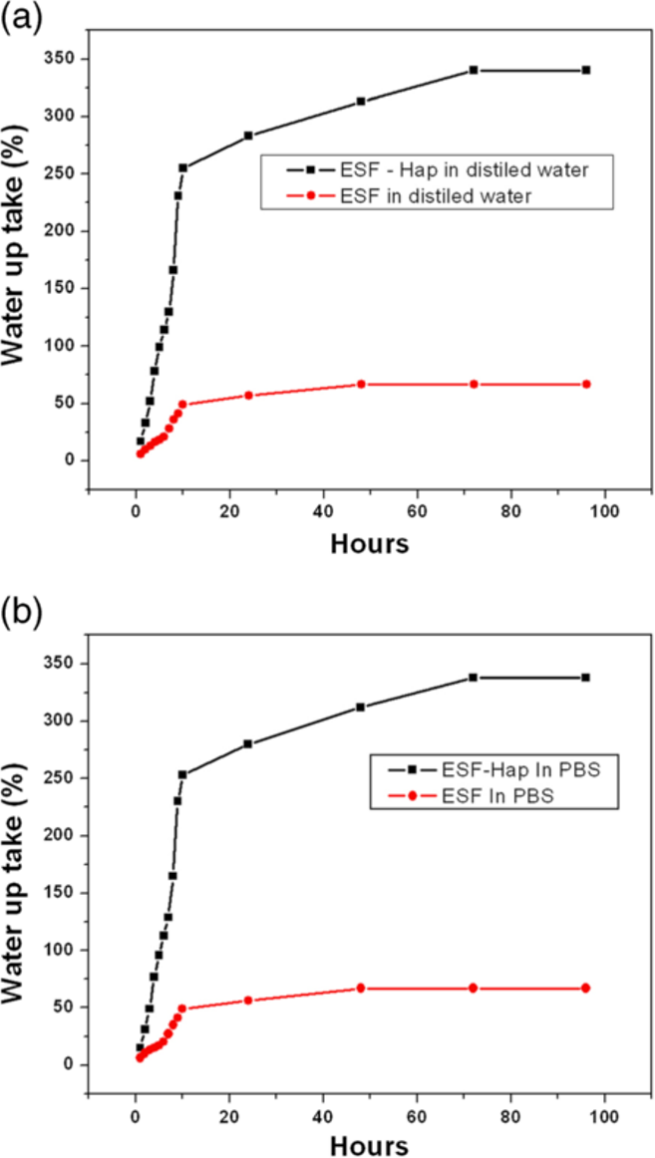
**Water uptake percentage of pure ESF and ESF-Hap scaffold in (a) distilled water and (b) PBS.**

The water-binding ability of the scaffold is an important function of biomaterials used for tissue culture applications. Figure 6a,b shows the water uptake percentage of the pure ESF and ESF-Hap scaffolds in distilled water and PBS, respectively. The figures show that the water uptake saturates at 96 h. The water uptake percentages of the pure ESF and ESF-Hap scaffolds in distilled water are 67% and 340% respectively in the saturated condition after 96 h. The water uptake percentages of the pure ESF and ESF-Hap scaffolds in PBS are 67% and 338% respectively after 96 h. The water uptake percentage of the ESF-Hap scaffold is higher in both distilled water and PBS than that of the pure ESF scaffold. Ito et al. (2005) found that hydroxyapatite has improved the hydrophilicity of poly(-3-hydroxyvalerate) scaffold (Ito et al.3-hydroxybutyrate-co 2005). Again, the water absorbency results show that the ESF-Hap scaffold could be a better biomaterial compared to the pure ESF scaffold.

### Biological studies of the pure ESF and ESF-Hap scaffolds

#### Bioactivity with SBF

**Figure 7:**
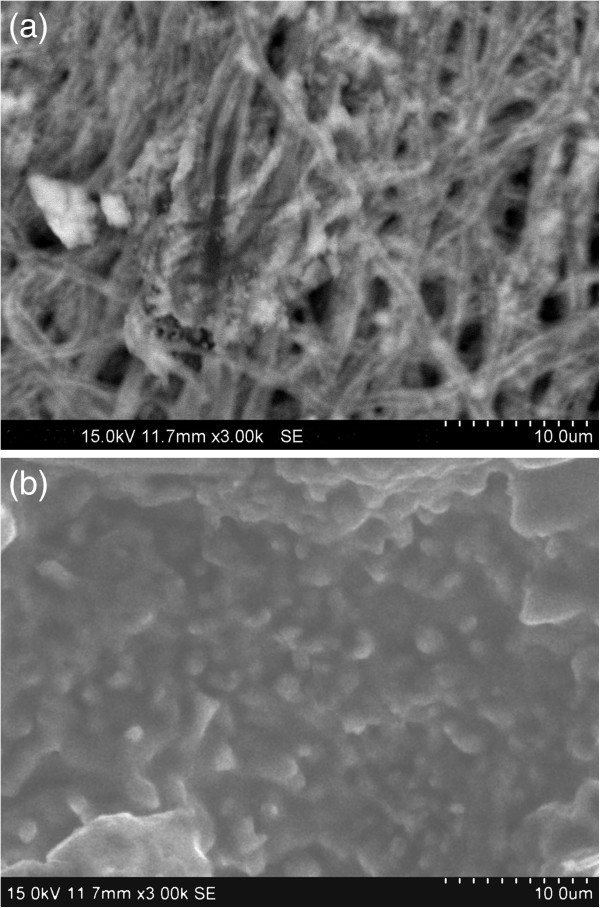
**Scaffold bioactivity in SBF.** (**a**) Pure ESF scaffold in SBF. (**b**) ESF-Hap scaffold in SBF after 15 days.

SEM images (Figure 7a,b) show respectively the pure ESF and ESF-Hap scaffolds in SBF. Figure 7b shows the deposition of hydroxyapatite crystals on the ESF-Hap scaffold, whereas the pure ESF scaffold shows the deposition of salts. It is observed from the SEM image that the hydroxyapatite crystal growth has covered the surface of the ESF-Hap scaffold. It promotes the formation of an irregular spherical aggregate on the scaffold, and this property may be exhibited on bone growth also (Ren et al. 2007).

#### Hemolysis

The hemocompatibility of the pure ESF and ESF-Hap scaffolds was evaluated by hemolysis test. The hemolysis percentage represents the extent of the red blood cells that hemolyzed when they come in contact with the sample. When the polymeric scaffold makes contact with blood, it must not induce thrombosis, thromboembolisms, antigenic responses, and destruction of blood constituents and plasma proteins. The positive reference is 100% hemolytic, and the negative reference is 0%. Table 1 shows that the hemolysis percentage of the pure ESF as well as ESF-Hap scaffolds are 1.22%. Both ESF and ESF-Hap scaffolds have hemolysis percentage less than 5% (Dhandayuthapani et al. 2012; Wang et al. 2010) which indicate that both pure ESF and ESF-Hap scaffolds exhibit good biocompatibility and may be suitable for biomaterials for clinical implant purposes.

**Table 1:** **Hemolysis percentage of pure eri silk fibroin scaffold and ESF-Hap scaffold**

Samples	Average OD value	Hemolysis (%)
Pure eri silk scaffold	0.056	0.850
ESF-Hap scaffold	0.056	0.765
Negative	0.055	0
Positive	0.137	100

#### Platelet adhesion

**Figure 8:**
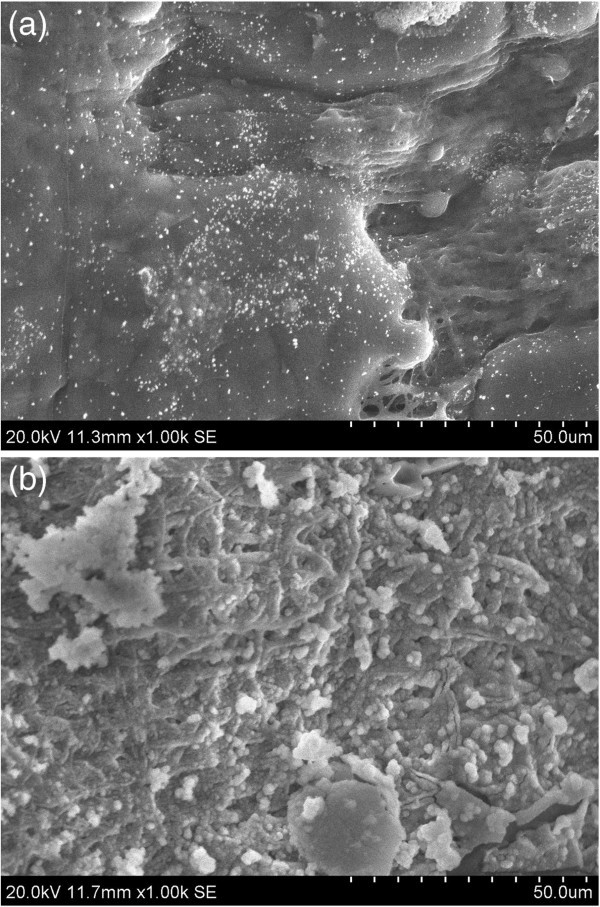
**SEM images of platelet adhesion on (a) pure ESF and (b) ESF-Hap scaffolds.**

SEM images given in Figure 8a,b show respectively the platelet adhesion on the pure ESF and ESF-Hap scaffolds after 1 h. The platelets, when incubated with the scaffold for an hour, stick to the surface of the scaffold, and their shape was not altered. The platelets retained their regular shape. The result also shows that the scaffold did not support the formation of a blood clot; the scaffold is blood compatible and may be used for biomedical applications (Dhandayuthapani et al. 2012).

#### hMSC attachment on the ESF and ESF-Hap scaffold

**Figure 9:**
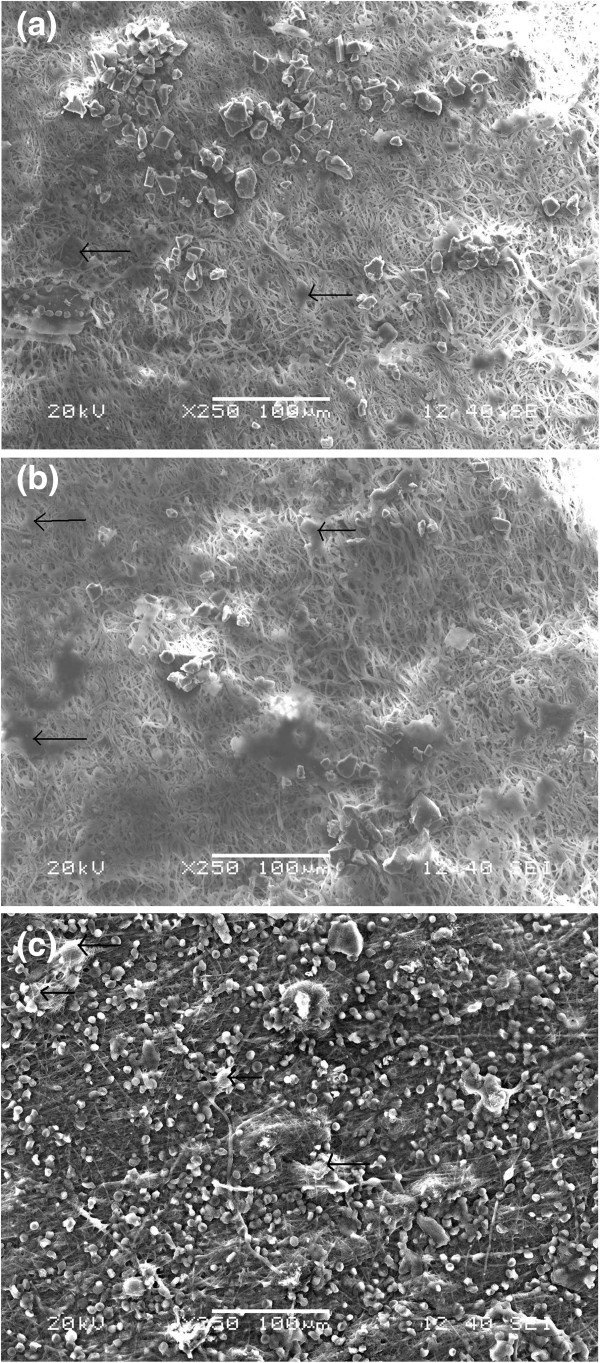
**SEM images of hMSC attachment on pure ESF scaffolds.** After (**a**) 3, (**b**) 5, and (**c**) 7 days.

**Figure 10:**
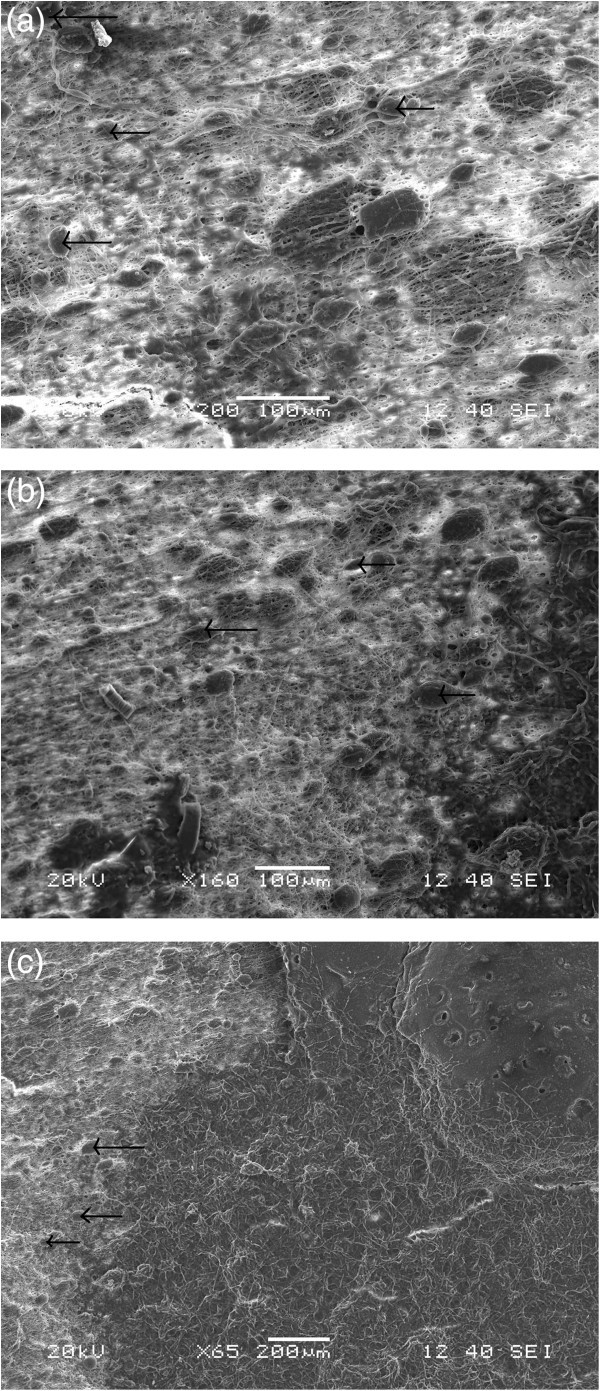
**SEM images of hMSC attachment on ESF-Hap scaffold.** After (**a**) 3, (**b**) 5, and (**c**) 7 days.

The SEM images in Figures 9a,b,c and 10a,b,c show the hMSC attachment and proliferation on the pure ESF and ESF-Hap scaffolds after 3, 5, and 7 days of incubation, respectively. The cell attachment and proliferation were found to have increased with the increase in incubation period. Moreover, after 3 days of incubation, the morphology of the cells changes to a spindle shape, which may indicate its differentiation. The cell attachment and profileration were found to be better in the ESF-Hap scaffold compared to the pure ESF because of the higher surface roughness and hydrophilicity of the ESF-Hap scaffold than that of the pure ESF scaffold.

#### Cell colonization and proliferation

**Figure 11:**
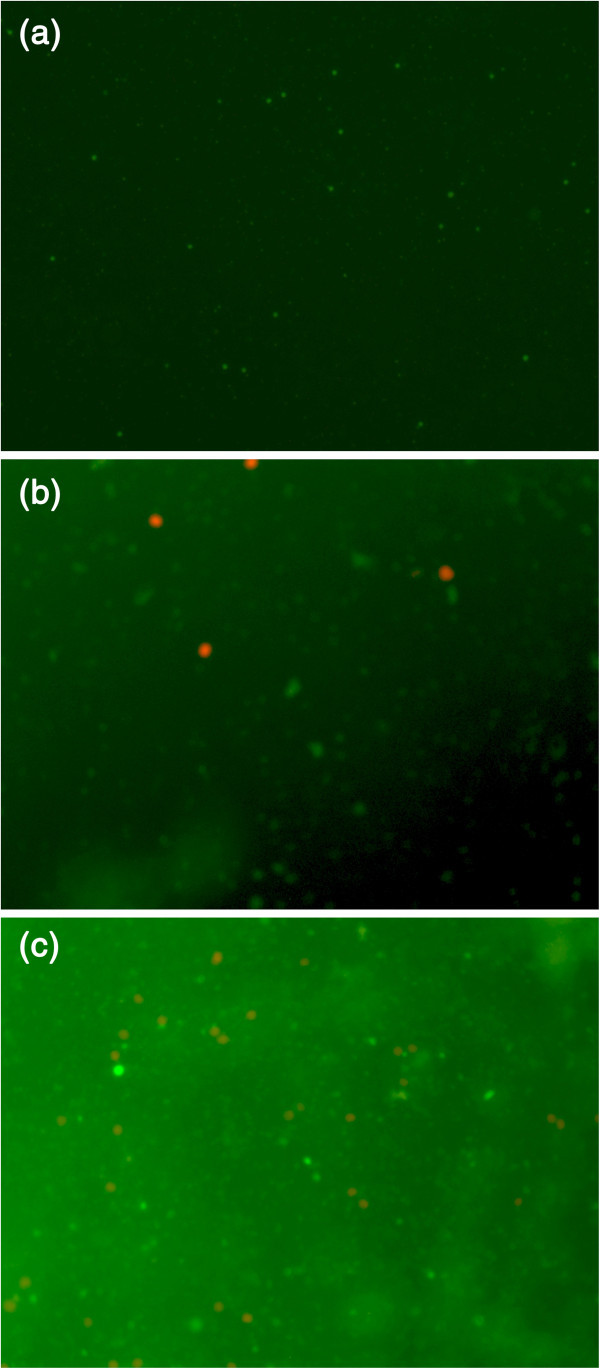
**Fluorescence image of cell adhesion on pure ESF scaffold.** After (**a**) 3, (**b**) 5, and (**c**) 7 days.

**Figure 12:**
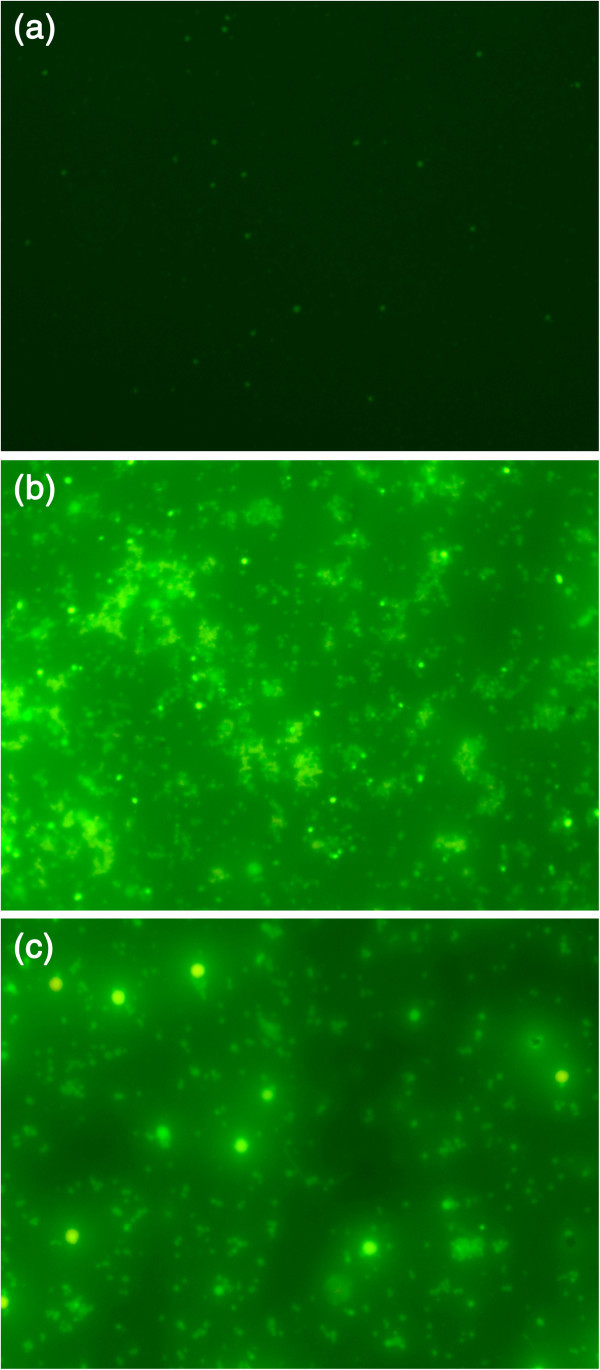
**Fluorescence image of cell adhesion on ESF-Hap scaffold.** After (**a**) 3, (**b**) 5, and (**c**) 7 days.

The hMSC viability study was carried out for both pure ESF and ESF-Hap scaffolds for a period of 7 days. The *in vitro* cell attachments of the two scaffolds were analyzed at three different time points of 3, 5, and 7 days. The cell colonization and proliferation were observed with respect to the incubation time. The fluorescence images in Figures 11a,b,c and 12a,b,c indicate the increase in the number of viable cells with an increase in the incubation period. A comparative study of the pure ESF and ESF-Hap scaffold shows increased number of cells in the ESF-Hap scaffold than in the pure ESF scaffold. The result proves that the hydroxyapatite favors the cell growth.

#### MTT assay

**Figure 13:**
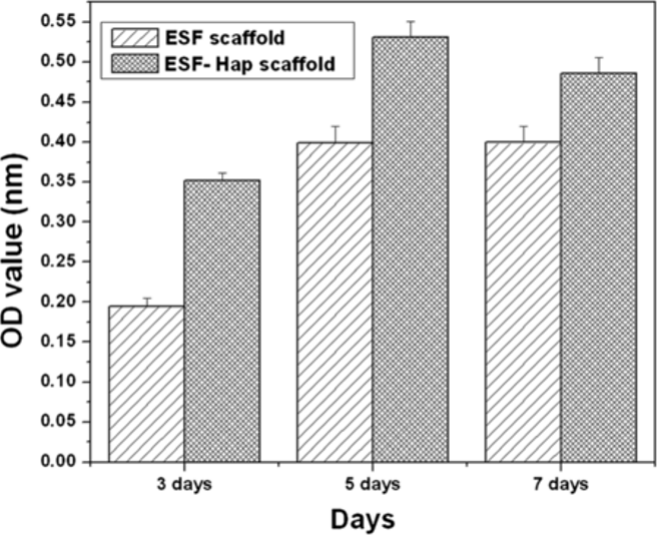
**MTT assay for pure ESF and ESF-Hap scaffolds.**

Figure 13 shows the percentage of the hMSC growth on the ESF and ESF-Hap scaffolds. The results were determined by measuring the optical density. The results have been shown for the hMSCs at different time points over a period of 7 days. For all the samples, cells continued to proliferate with the increase in culture time. After culturing for 7 days, the cell numbers increased considerably, compared to those cultured for 3 days. Furthermore, after 3 days, the cells on the ESF-Hap scaffold proliferated more actively, compared to those on the pure ESF scaffold, indicating that Hap promoted the proliferation of cells. The results suggest that the bioactive Hap phase might play an important role in mediating cellular response to the composites.

## Conclusions

Eri silk fibroin solution was spun into a scaffold by electrospinning. The scaffold was treated with hydroxyapatite to evaluate its suitability for bone tissue engineering applications. The composite of ESF and hydroxyapatite scaffolds were synthesized by alternate soaking of the eri silk fibroin scaffold in calcium chloride and sodium diammonium phosphate. The following conclusions were made from the physical characterization of the scaffolds:

- Majority of the electrospun pure ESF fibers have a diameter in the range of 600 to 800 nm, and the diameter is 1,000 to 1,200 nm for the fibers present in ESF-Hap scaffold.
- Porosity of the scaffolds is found to be in the range of 74% to 78%.
- Crystallinity and thermal stability of ESF-Hap scaffold are higher than that of pure ESF scaffold.
- Water uptake percentage of the ESF-Hap scaffold is more than that of the pure ESF scaffold.
- Tensile strength and stress of the ESF-Hap scaffold are less than those of the pure eri silk scaffold.

The above physical characteristics of the scaffold show that both the ESF and ESF-Hap scaffolds are suitable for tissue engineering applications.

The following conclusions are drawn from the biological characterization of the scaffolds:

- Both the ESF-Hap and pure ESF scaffolds are proved to be hemocompatible.
- Platelet aggregations and deformation are not found in both scaffolds.
- hMSC attachment and density of cells increase more on the ESF-Hap scaffold than on the pure ESF scaffold as the incubation time increased.
- Cell viability of the ESF-Hap scaffold is higher than that the of pure ESF scaffold.

The biological studies show that the ESF-Hap scaffold is a better material for bone tissue engineering applications compared to pure ESF scaffolds.

## Authors’ original submitted files for images

Below are the links to the authors’ original submitted files for images. Authors’ original file for figure 1 Authors’ original file for figure 2 Authors’ original file for figure 3 Authors’ original file for figure 4 Authors’ original file for figure 5 Authors’ original file for figure 6 Authors’ original file for figure 7 Authors’ original file for figure 8 Authors’ original file for figure 9 Authors’ original file for figure 10 Authors’ original file for figure 11 Authors’ original file for figure 12 Authors’ original file for figure 13 Authors’ original file for figure 14 Authors’ original file for figure 15 Authors’ original file for figure 16 Authors’ original file for figure 17 Authors’ original file for figure 18 Authors’ original file for figure 19 Authors’ original file for figure 20 Authors’ original file for figure 21 Authors’ original file for figure 22 Authors’ original file for figure 23 Authors’ original file for figure 24 Authors’ original file for figure 25 Authors’ original file for figure 26 Authors’ original file for figure 27 Authors’ original file for figure 28 Authors’ original file for figure 29
