# Supplementary figures and images for: Electrospun eri silk fibroin scaffold coated with hydroxyapatite for bone tissue engineering applications
Source: Prog Biomater. 2013 Mar 8;2:6. doi: 10.1186/2194-0517-2-6 (PMC5964657; doi:10.1186/2194-0517-2-6)

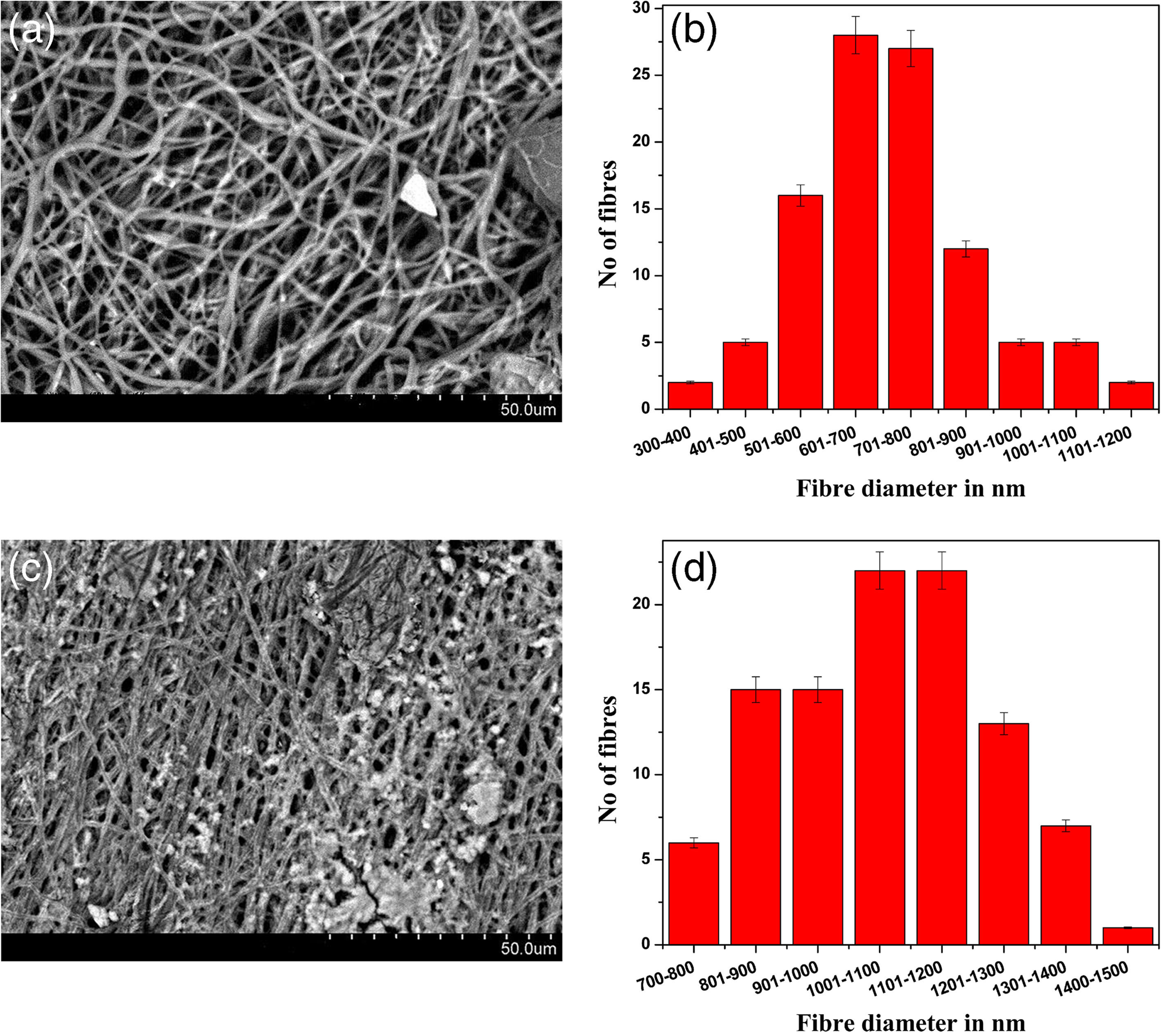

Supplement: Supplementary file 1 — Authors’ original file for figure 1 [file 40204_2012_15_MOESM1_ESM.tiff]

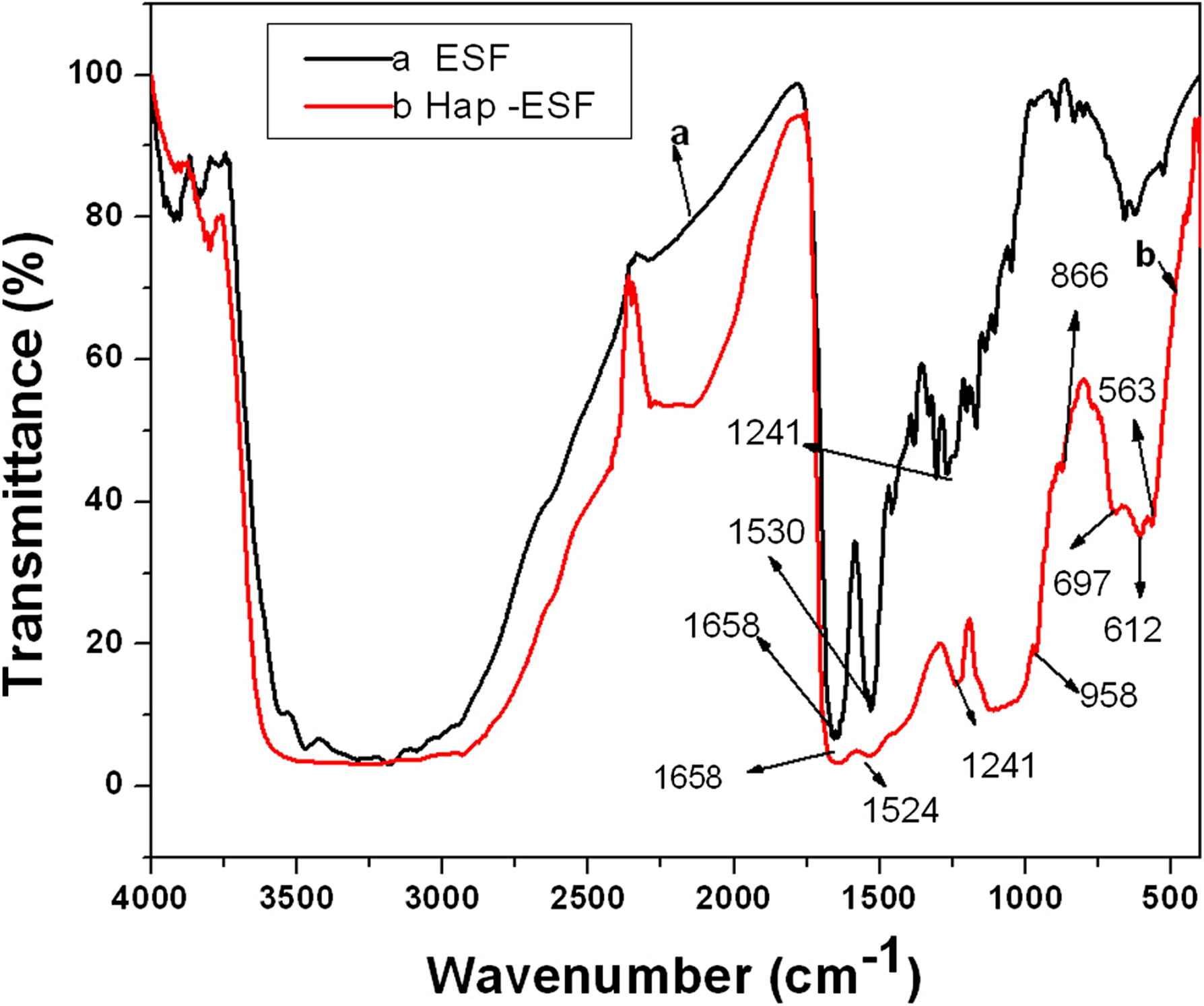

Supplement: Supplementary file 2 — Authors’ original file for figure 2 [file 40204_2012_15_MOESM2_ESM.tiff]

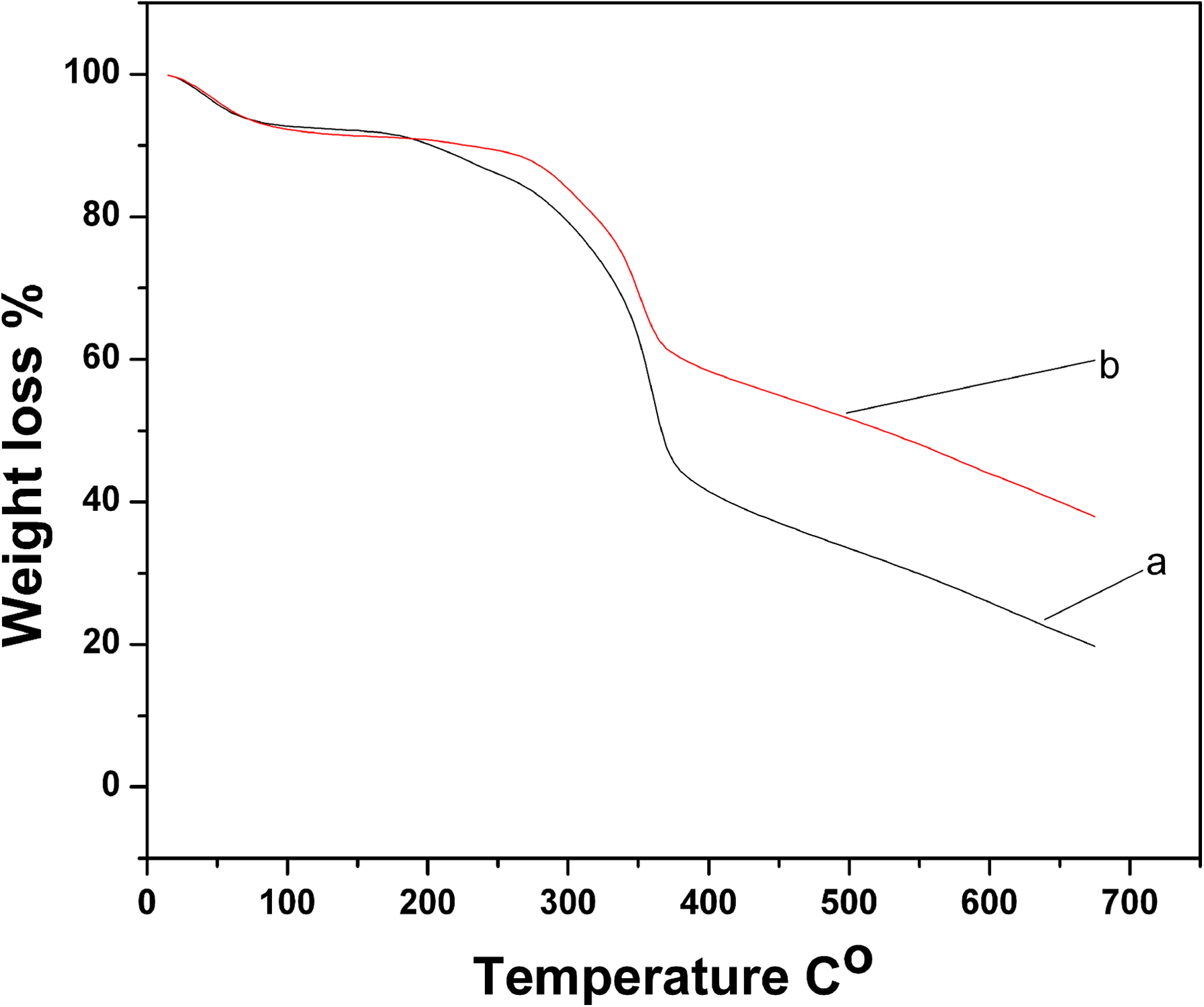

Supplement: Supplementary file 3 — Authors’ original file for figure 3 [file 40204_2012_15_MOESM3_ESM.tiff]

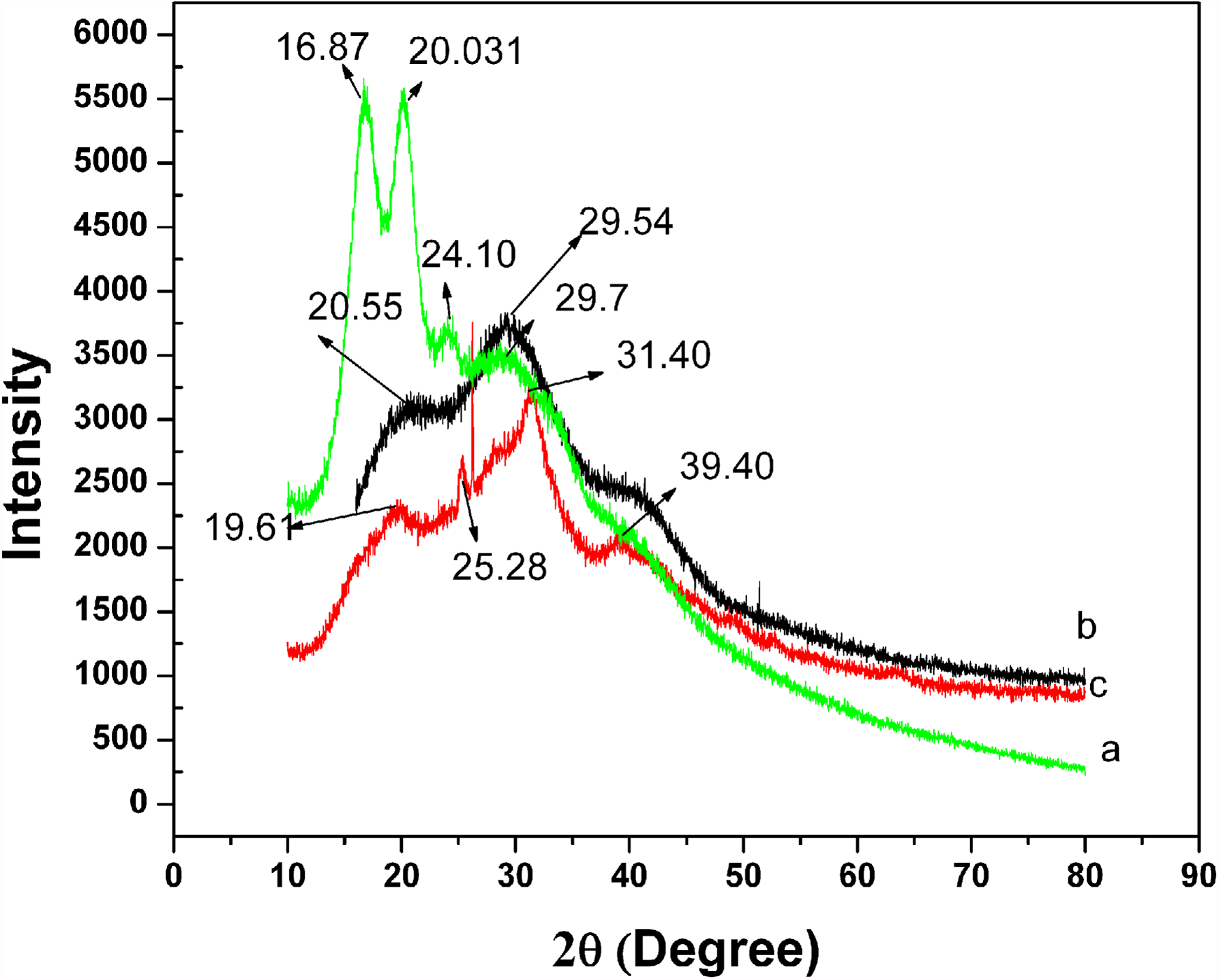

Supplement: Supplementary file 4 — Authors’ original file for figure 4 [file 40204_2012_15_MOESM4_ESM.tiff]

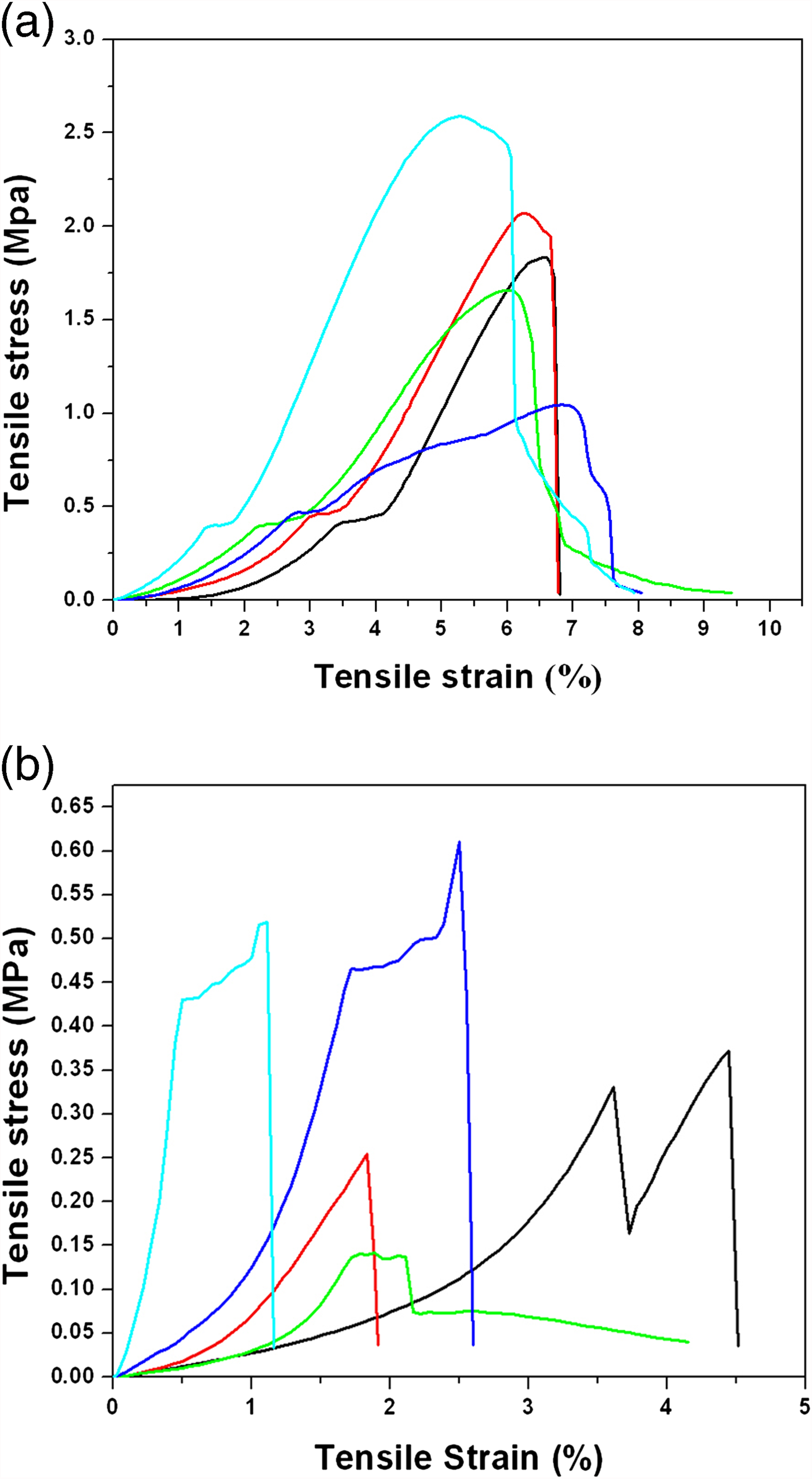

Supplement: Supplementary file 5 — Authors’ original file for figure 5 [file 40204_2012_15_MOESM5_ESM.tiff]

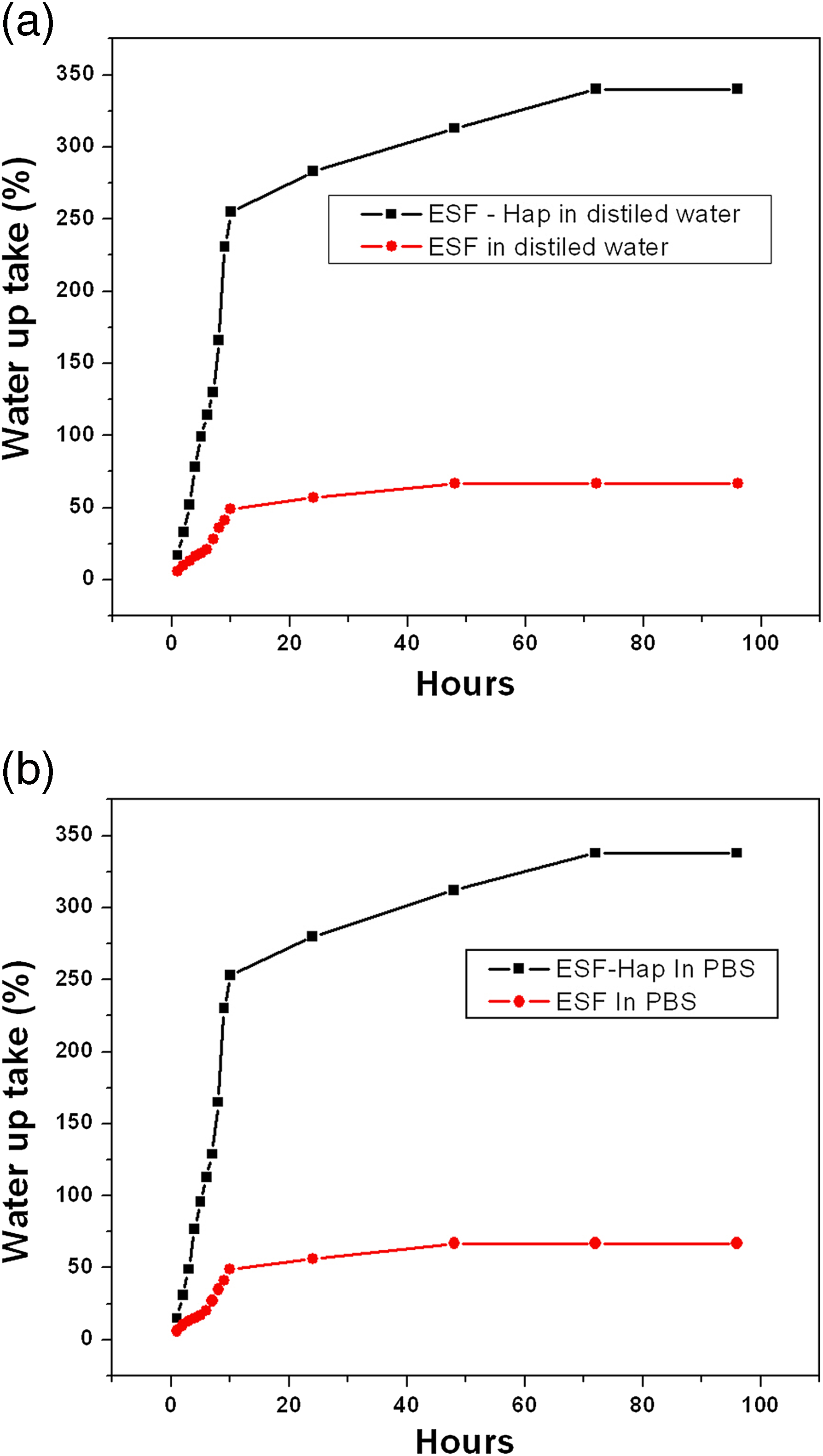

Supplement: Supplementary file 6 — Authors’ original file for figure 6 [file 40204_2012_15_MOESM6_ESM.tiff]

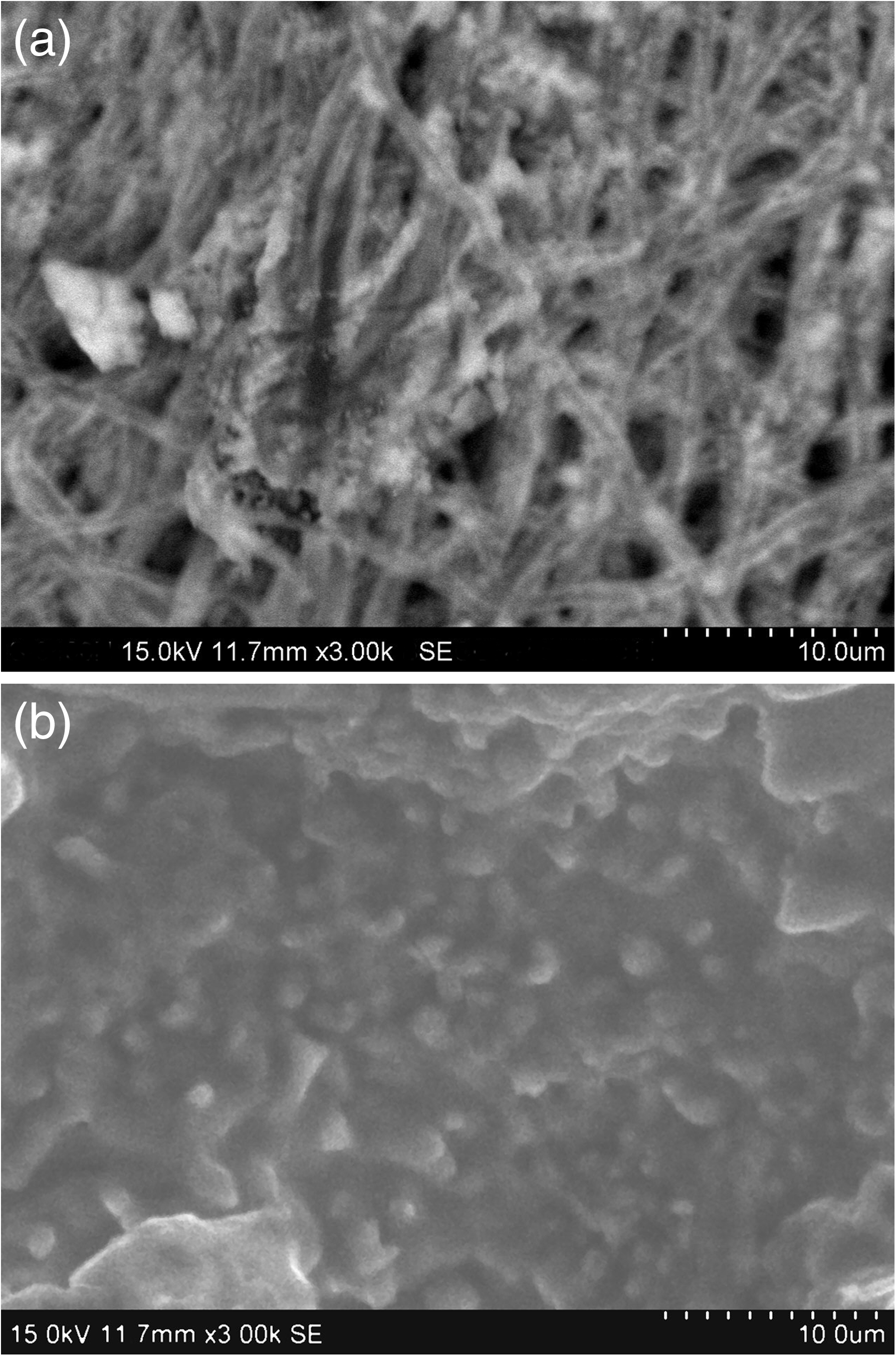

Supplement: Supplementary file 7 — Authors’ original file for figure 7 [file 40204_2012_15_MOESM7_ESM.tiff]

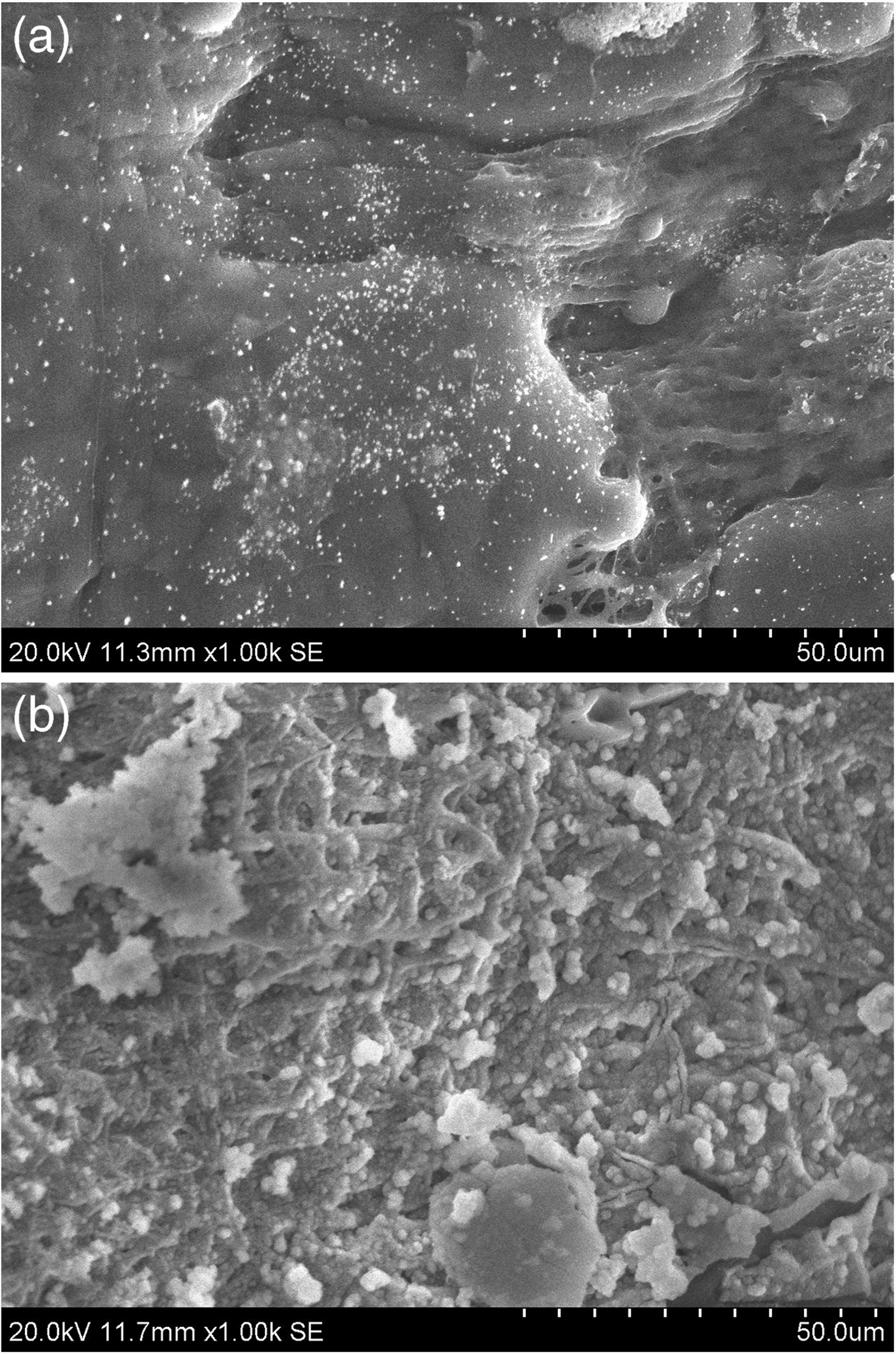

Supplement: Supplementary file 8 — Authors’ original file for figure 8 [file 40204_2012_15_MOESM8_ESM.tiff]

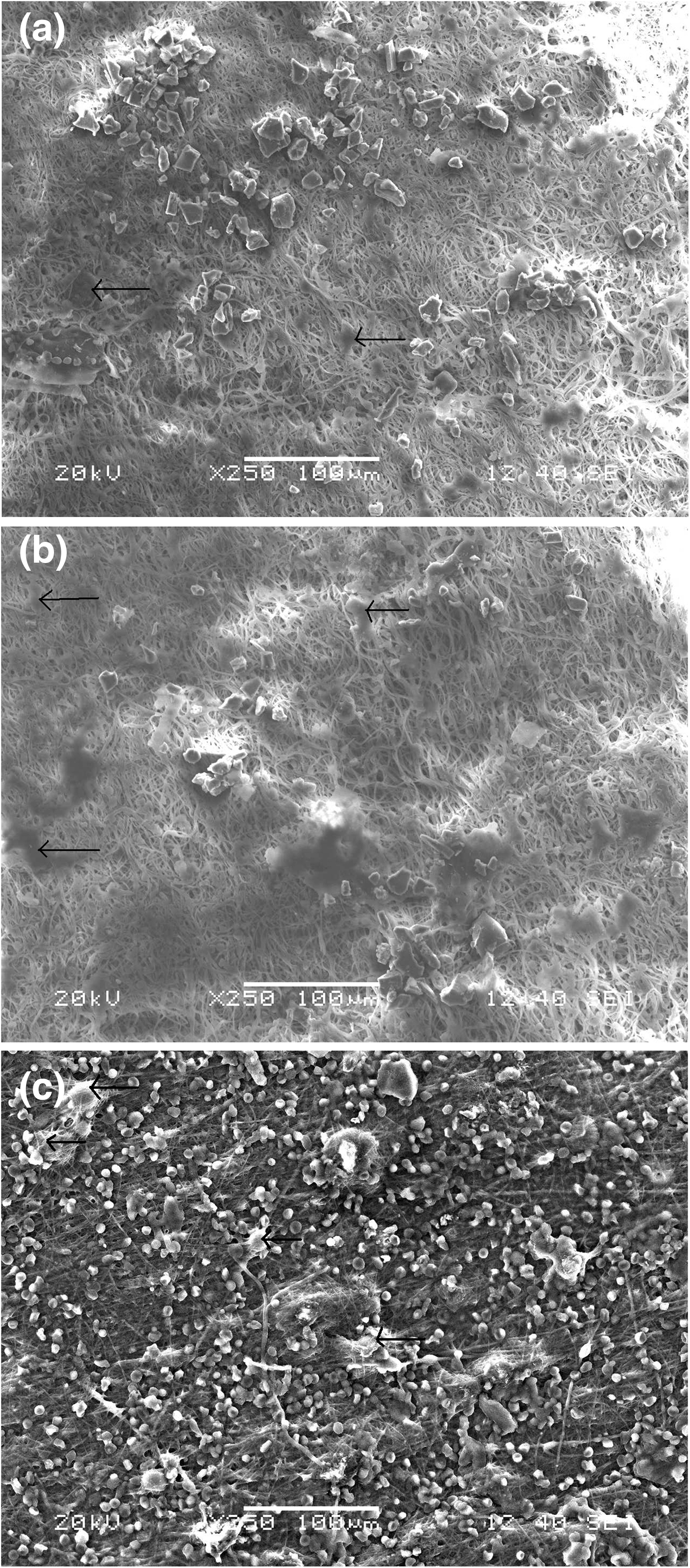

Supplement: Supplementary file 9 — Authors’ original file for figure 9 [file 40204_2012_15_MOESM9_ESM.tiff]

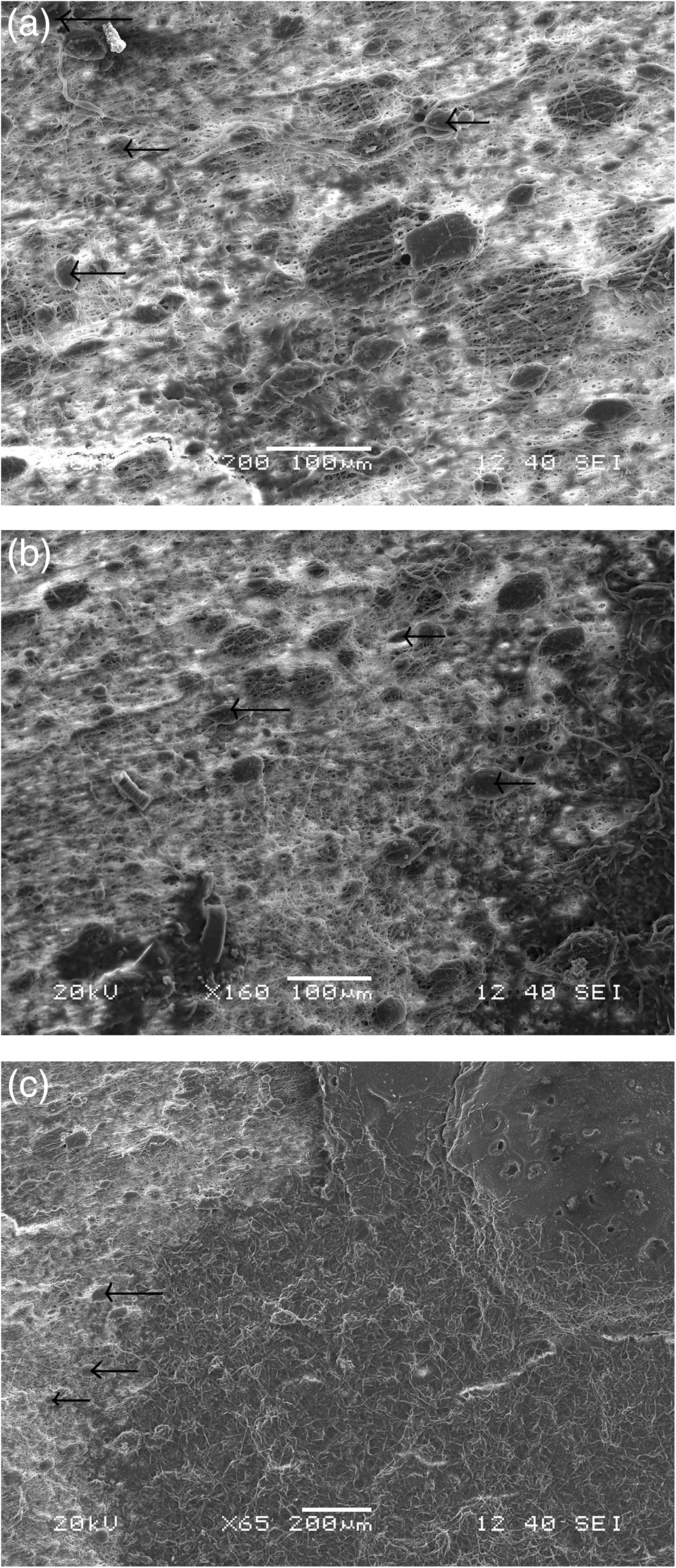

Supplement: Supplementary file 10 — Authors’ original file for figure 10 [file 40204_2012_15_MOESM10_ESM.tiff]

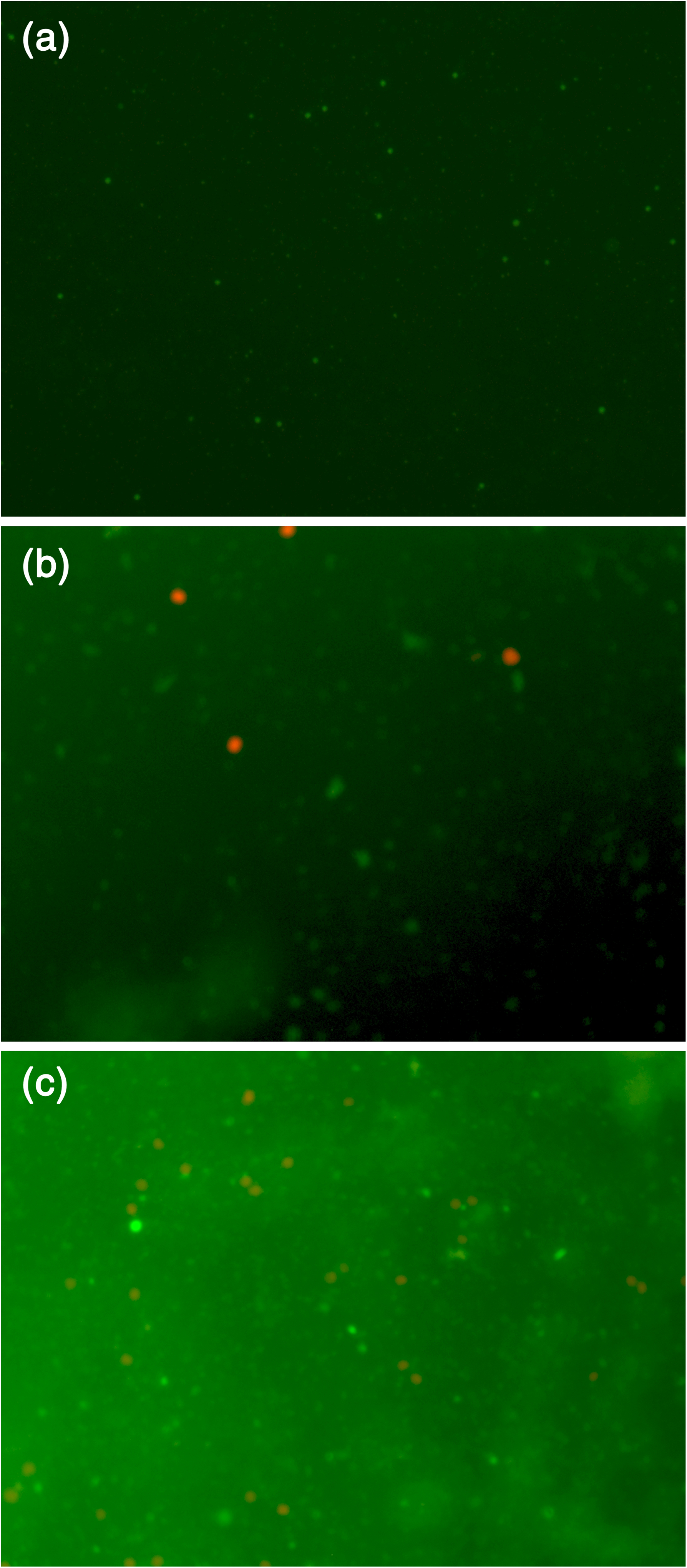

Supplement: Supplementary file 11 — Authors’ original file for figure 11 [file 40204_2012_15_MOESM11_ESM.tiff]

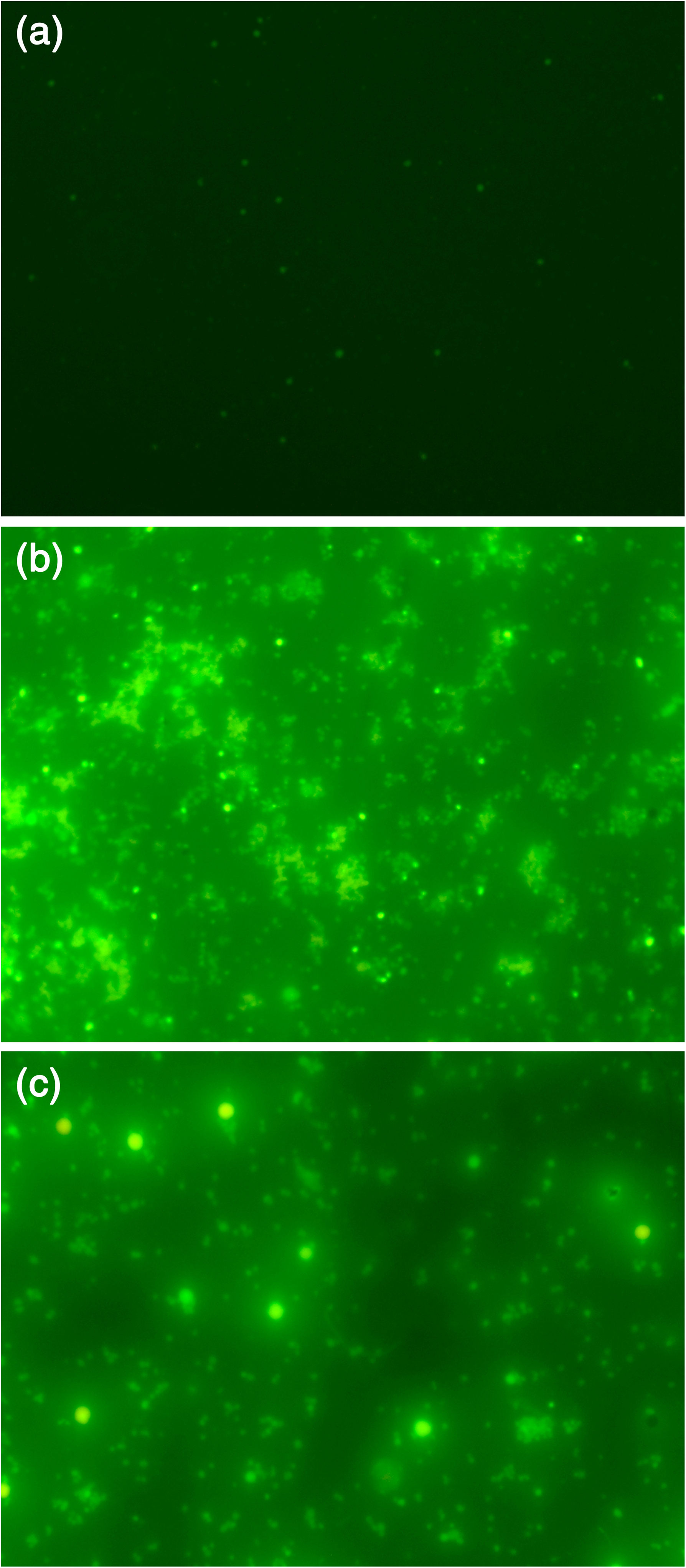

Supplement: Supplementary file 12 — Authors’ original file for figure 12 [file 40204_2012_15_MOESM12_ESM.tiff]

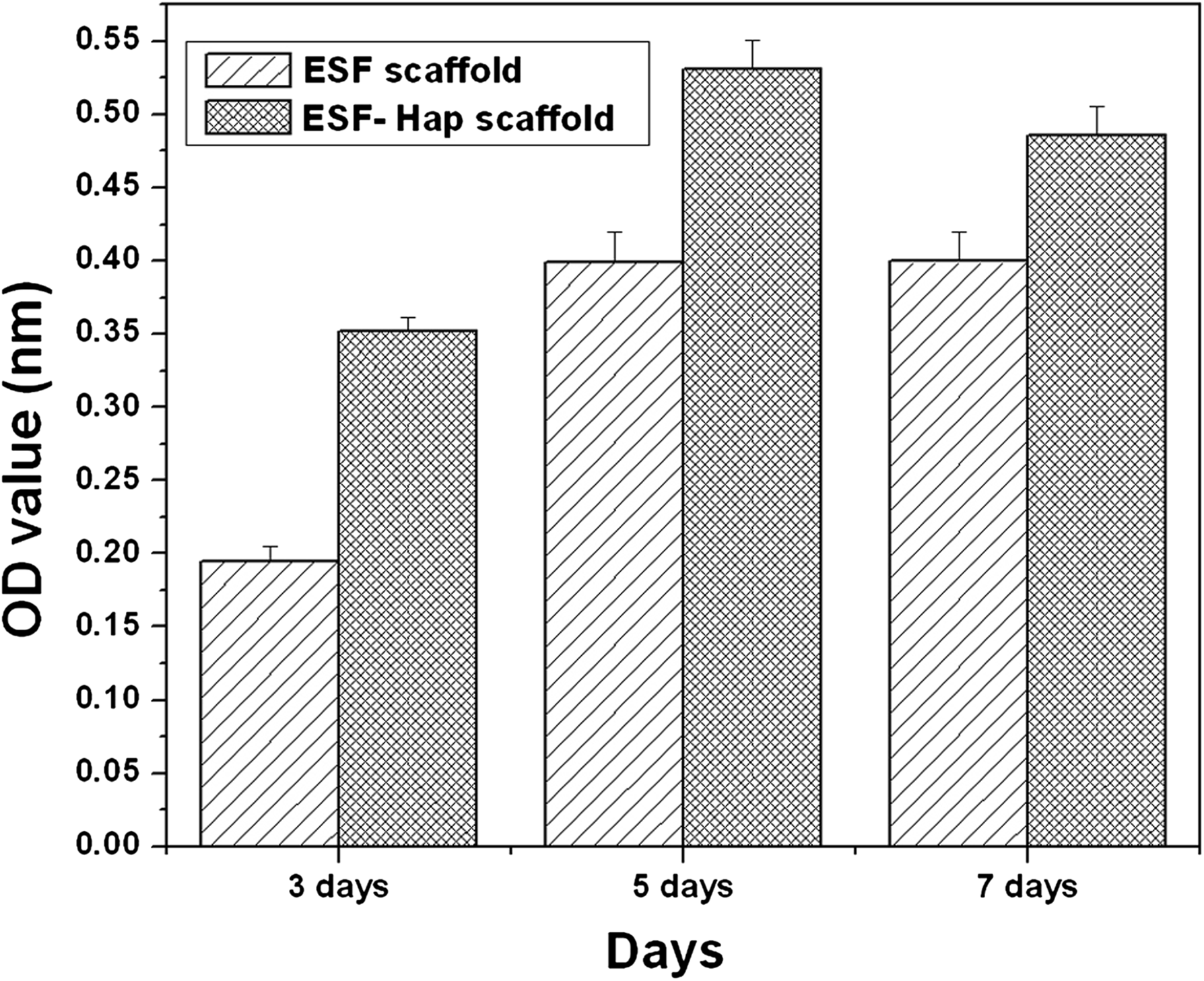

Supplement: Supplementary file 13 — Authors’ original file for figure 13 [file 40204_2012_15_MOESM13_ESM.tiff]

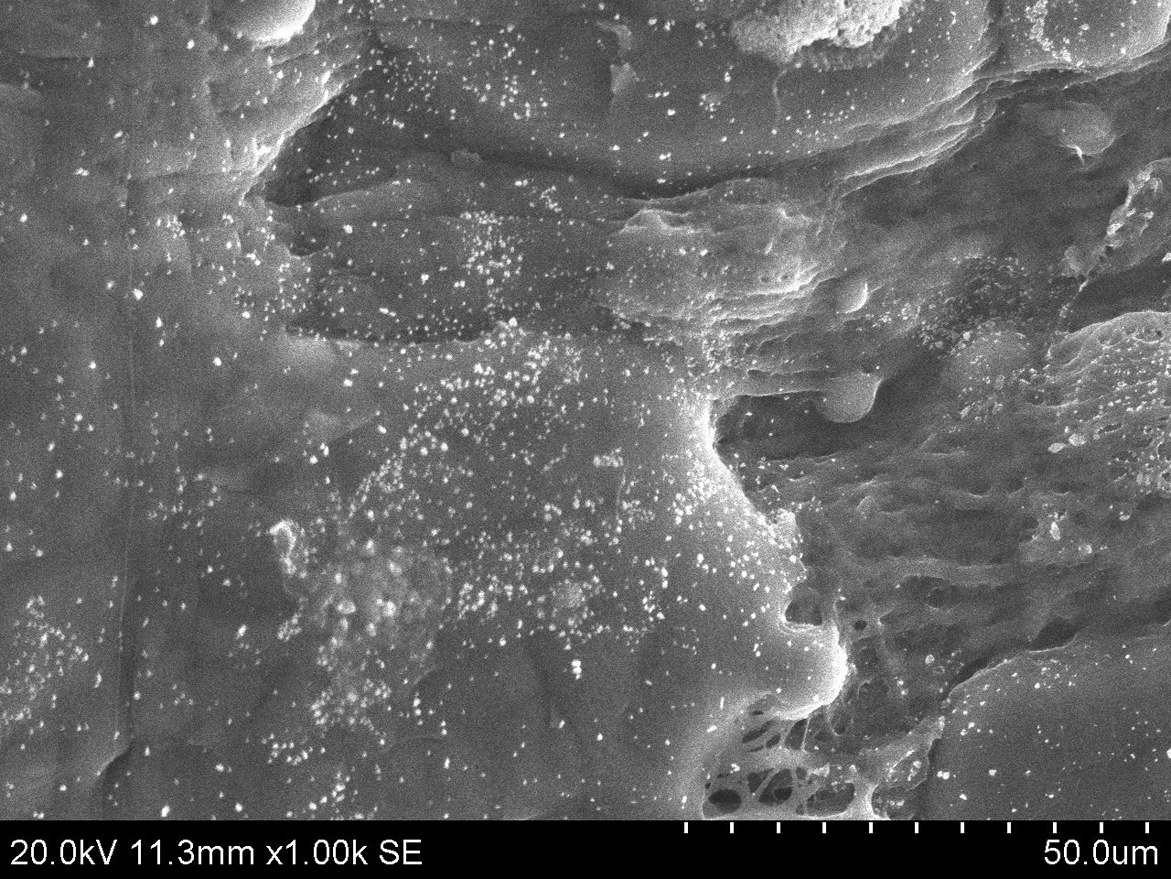

Supplement: Supplementary file 15 — Authors’ original file for figure 15 [file 40204_2012_15_MOESM15_ESM.tiff]

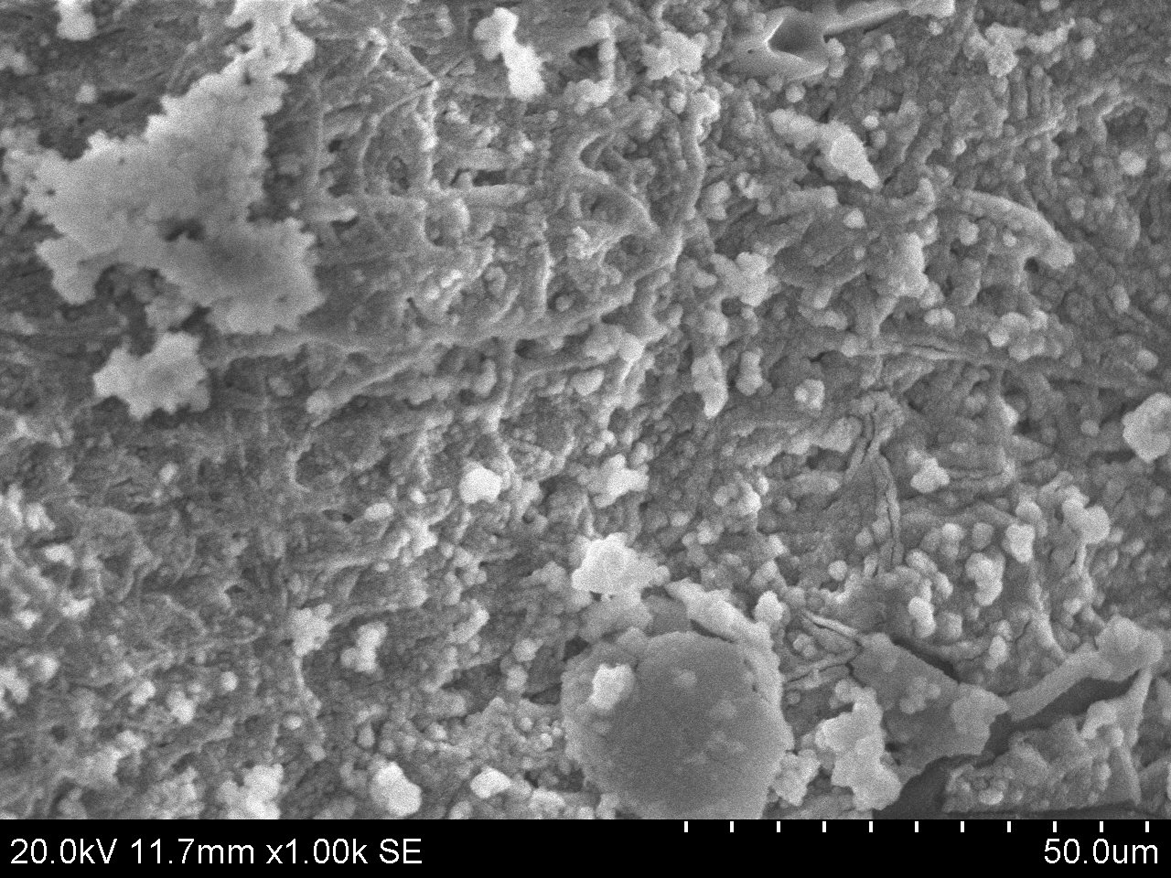

Supplement: Supplementary file 16 — Authors’ original file for figure 16 [file 40204_2012_15_MOESM16_ESM.tiff]

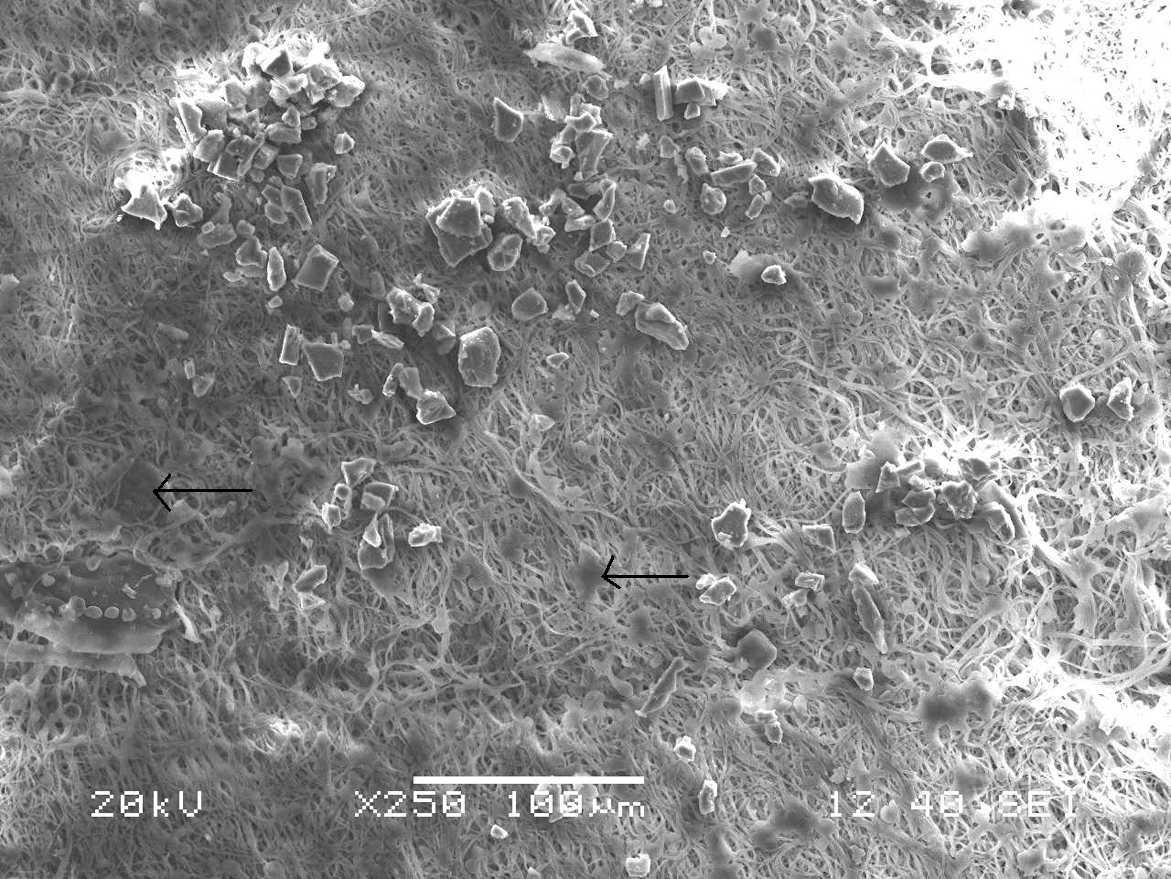

Supplement: Supplementary file 17 — Authors’ original file for figure 17 [file 40204_2012_15_MOESM17_ESM.tiff]

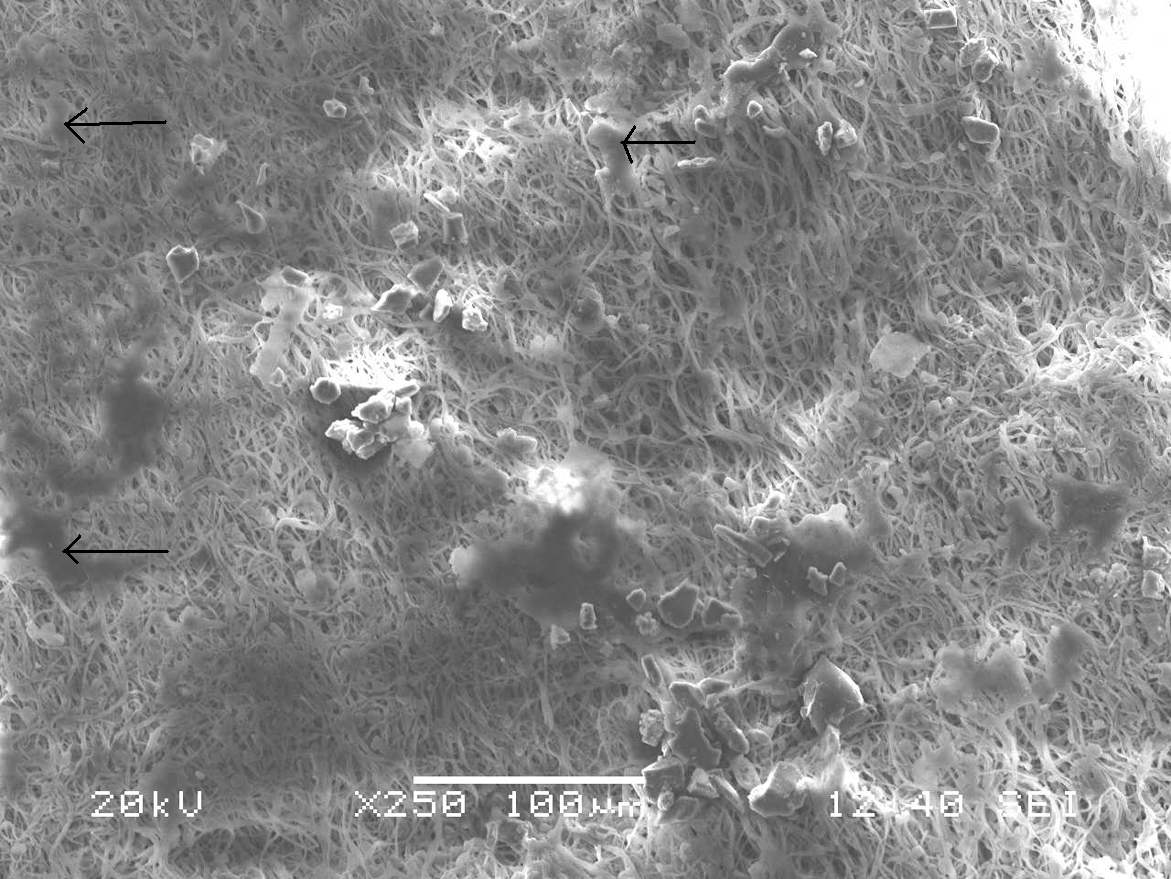

Supplement: Supplementary file 18 — Authors’ original file for figure 18 [file 40204_2012_15_MOESM18_ESM.tiff]

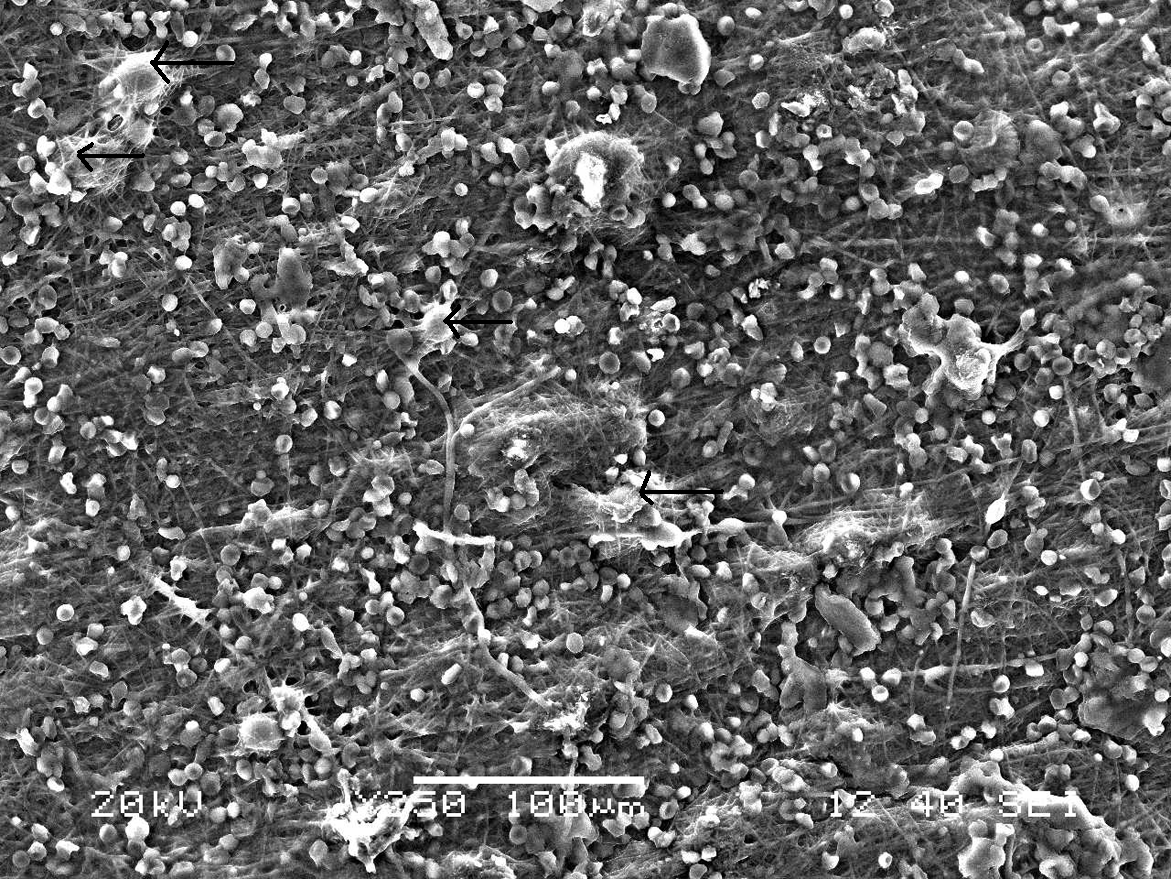

Supplement: Supplementary file 19 — Authors’ original file for figure 19 [file 40204_2012_15_MOESM19_ESM.tiff]

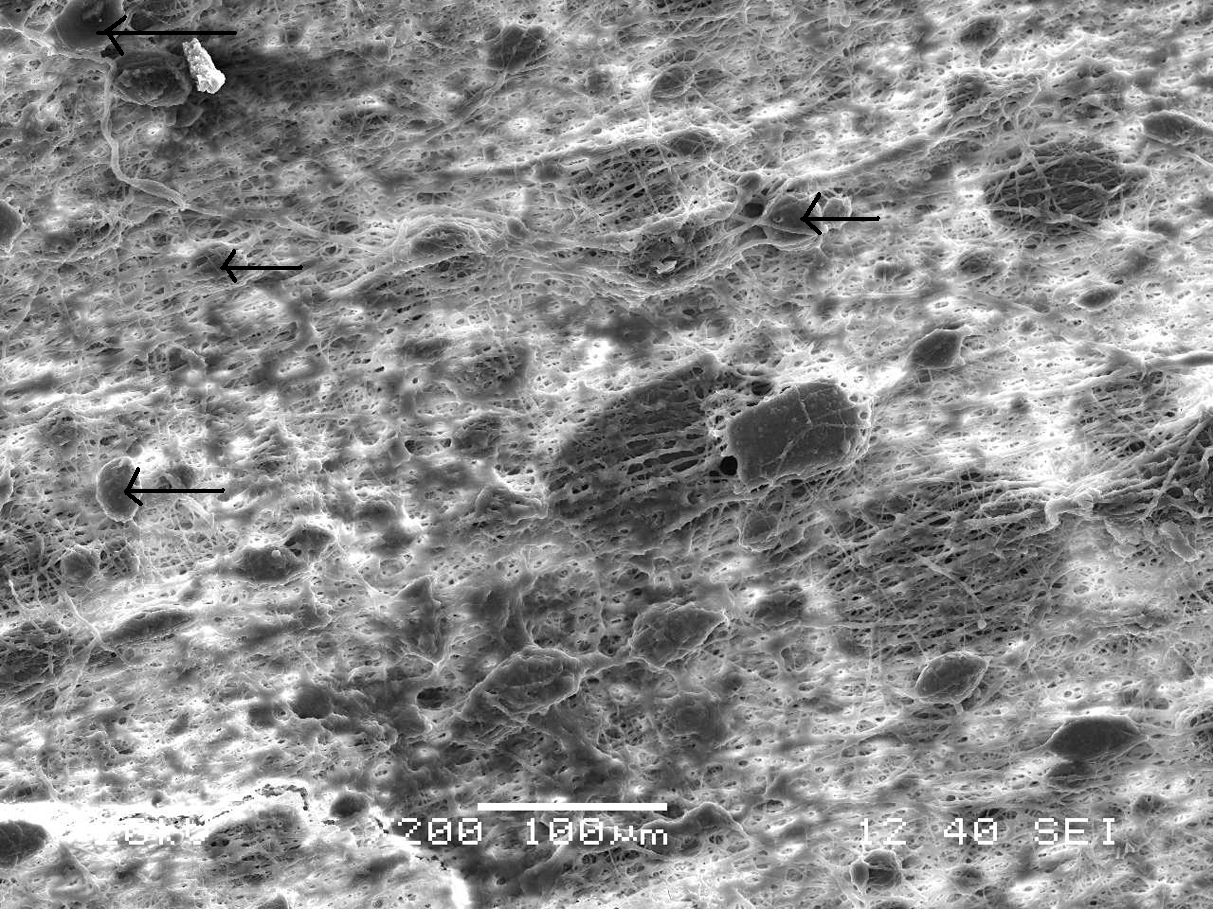

Supplement: Supplementary file 20 — Authors’ original file for figure 20 [file 40204_2012_15_MOESM20_ESM.tiff]

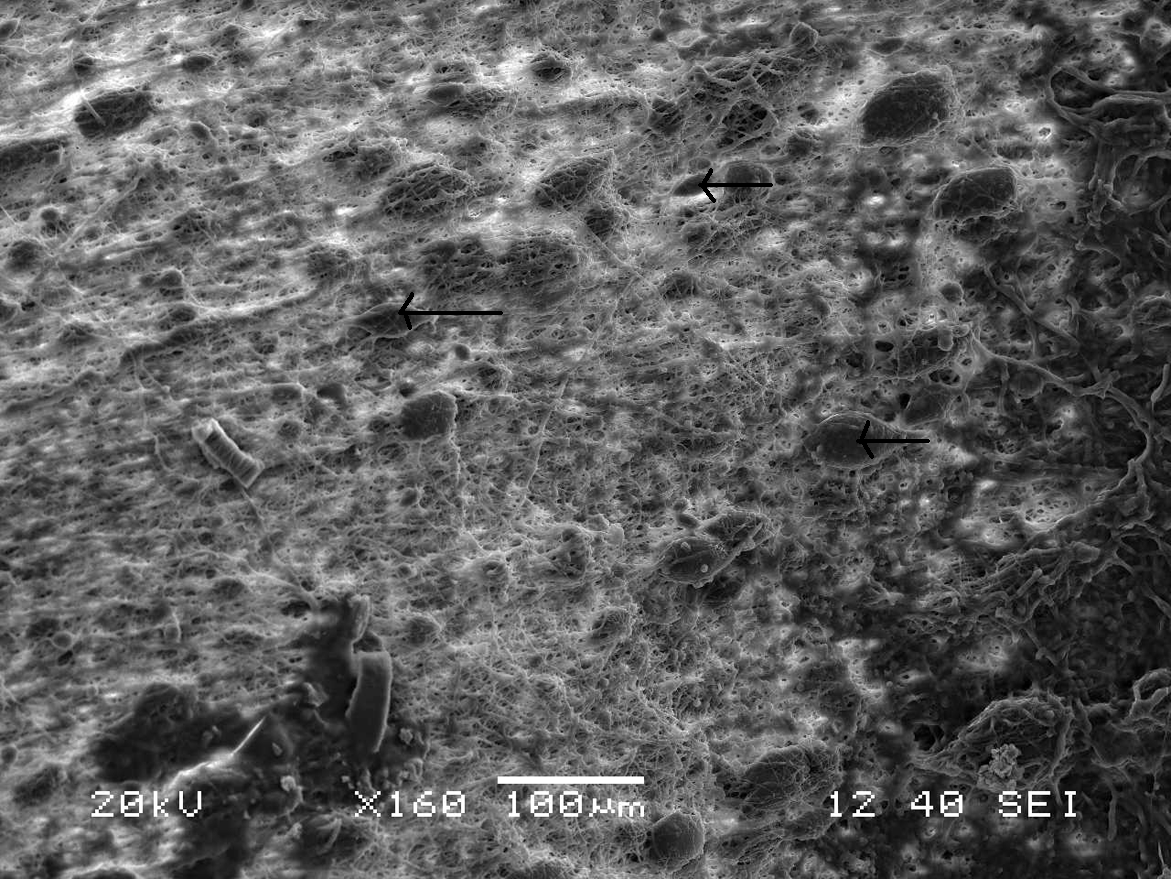

Supplement: Supplementary file 21 — Authors’ original file for figure 21 [file 40204_2012_15_MOESM21_ESM.tiff]

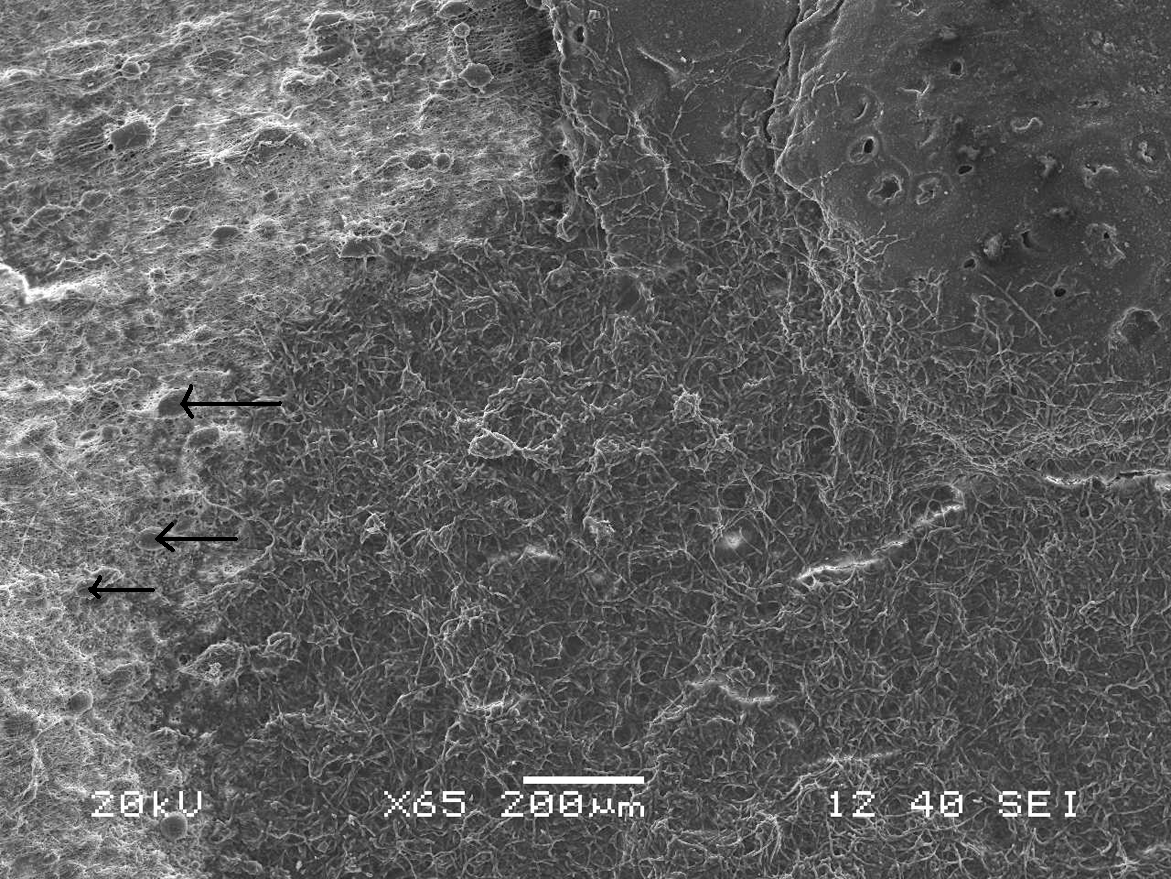

Supplement: Supplementary file 22 — Authors’ original file for figure 22 [file 40204_2012_15_MOESM22_ESM.tiff]

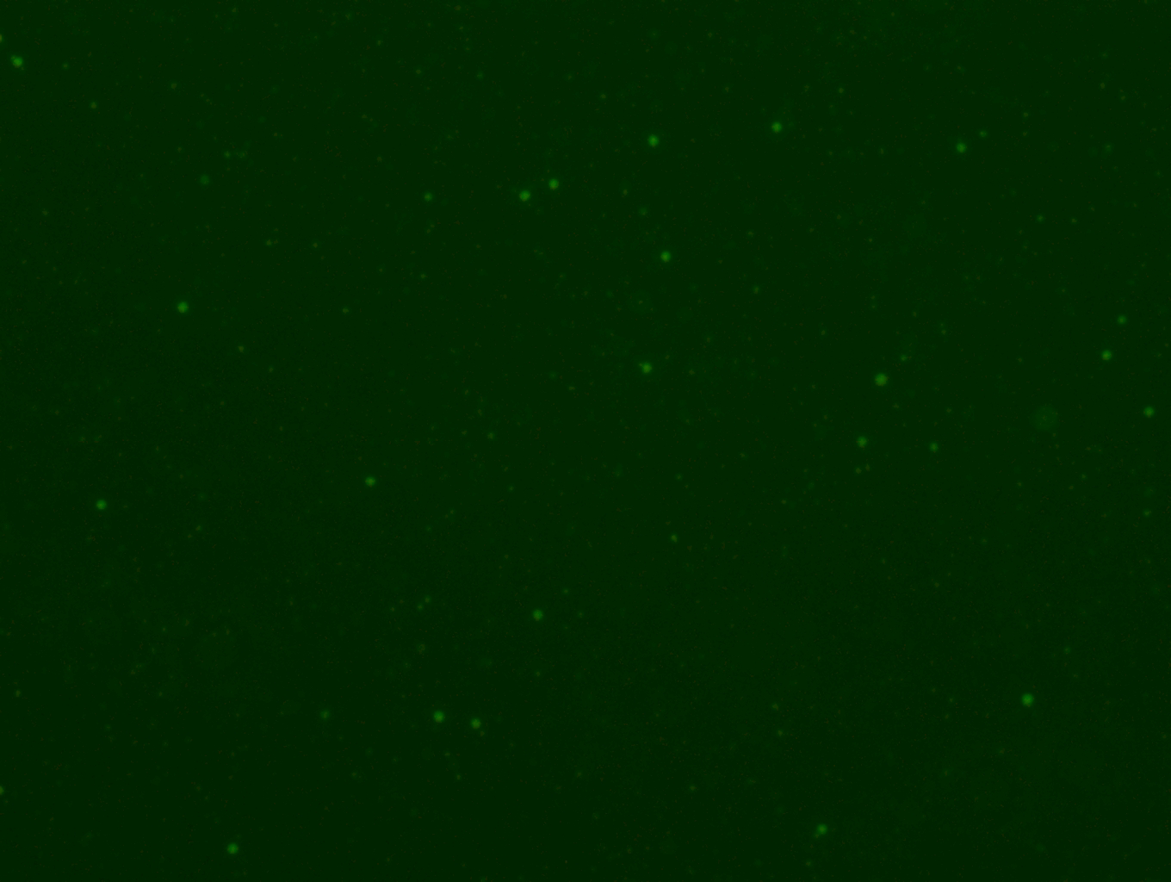

Supplement: Supplementary file 23 — Authors’ original file for figure 23 [file 40204_2012_15_MOESM23_ESM.tiff]

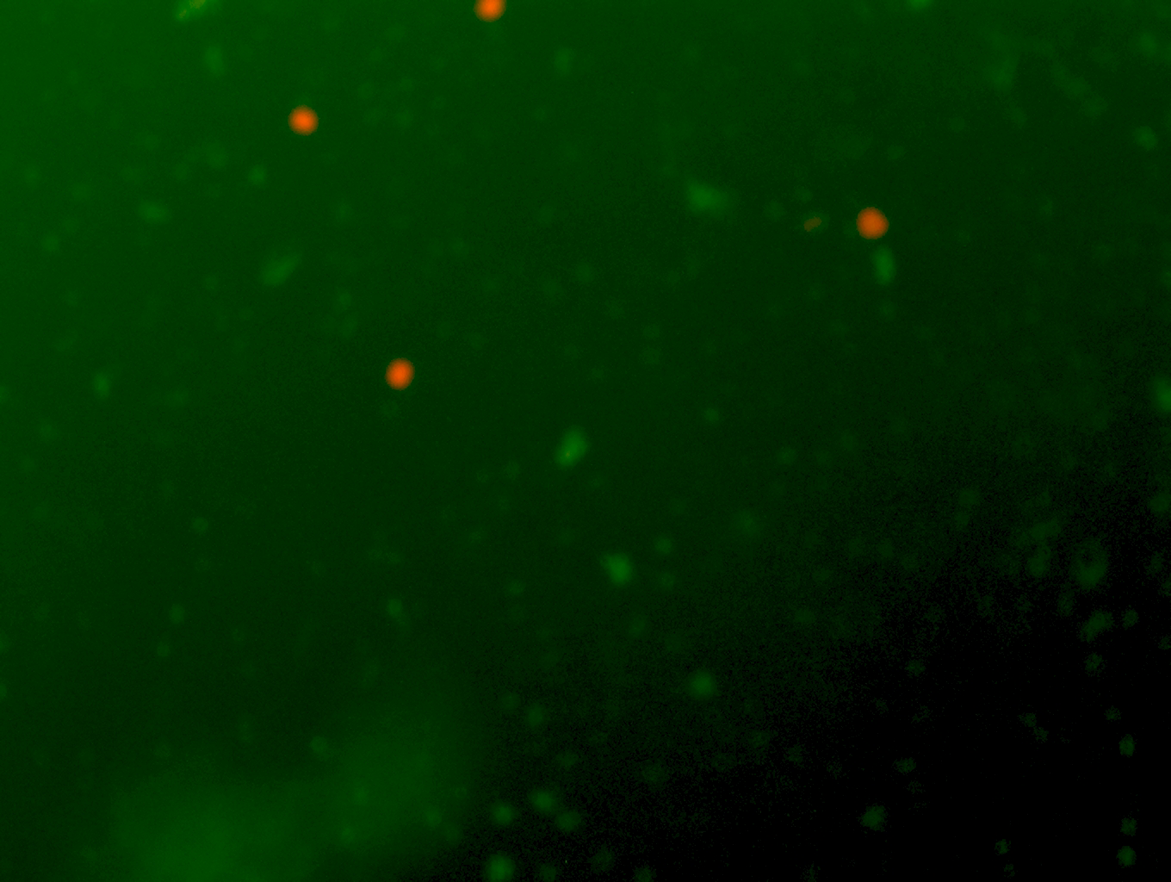

Supplement: Supplementary file 24 — Authors’ original file for figure 24 [file 40204_2012_15_MOESM24_ESM.tiff]

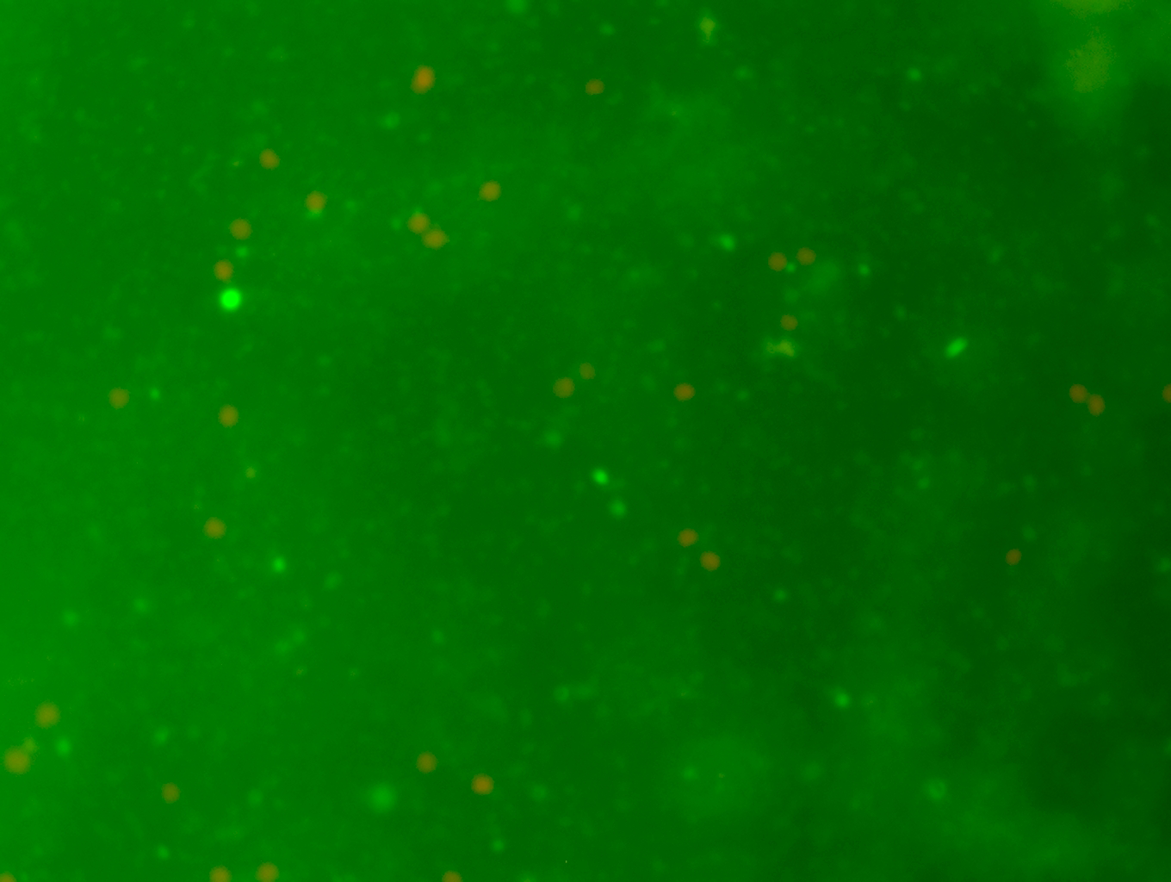

Supplement: Supplementary file 25 — Authors’ original file for figure 25 [file 40204_2012_15_MOESM25_ESM.tiff]

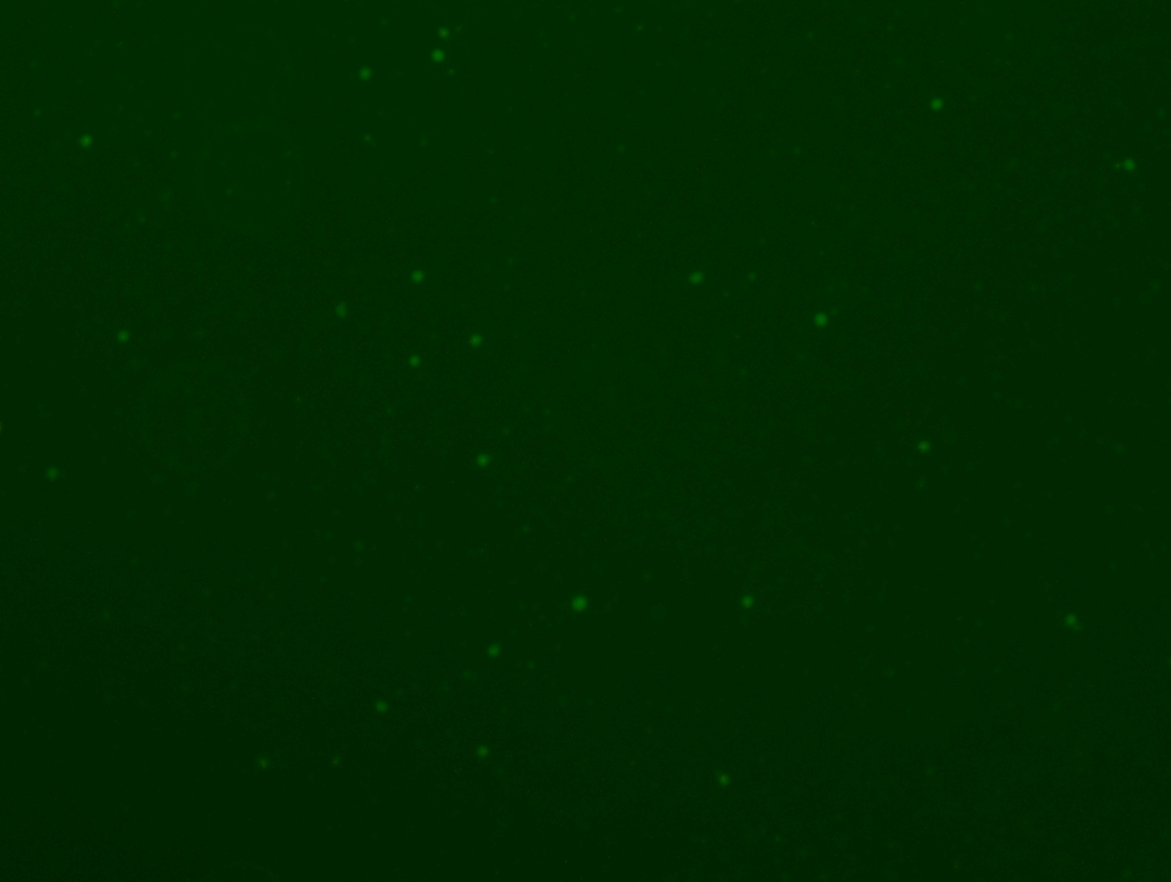

Supplement: Supplementary file 26 — Authors’ original file for figure 26 [file 40204_2012_15_MOESM26_ESM.tiff]

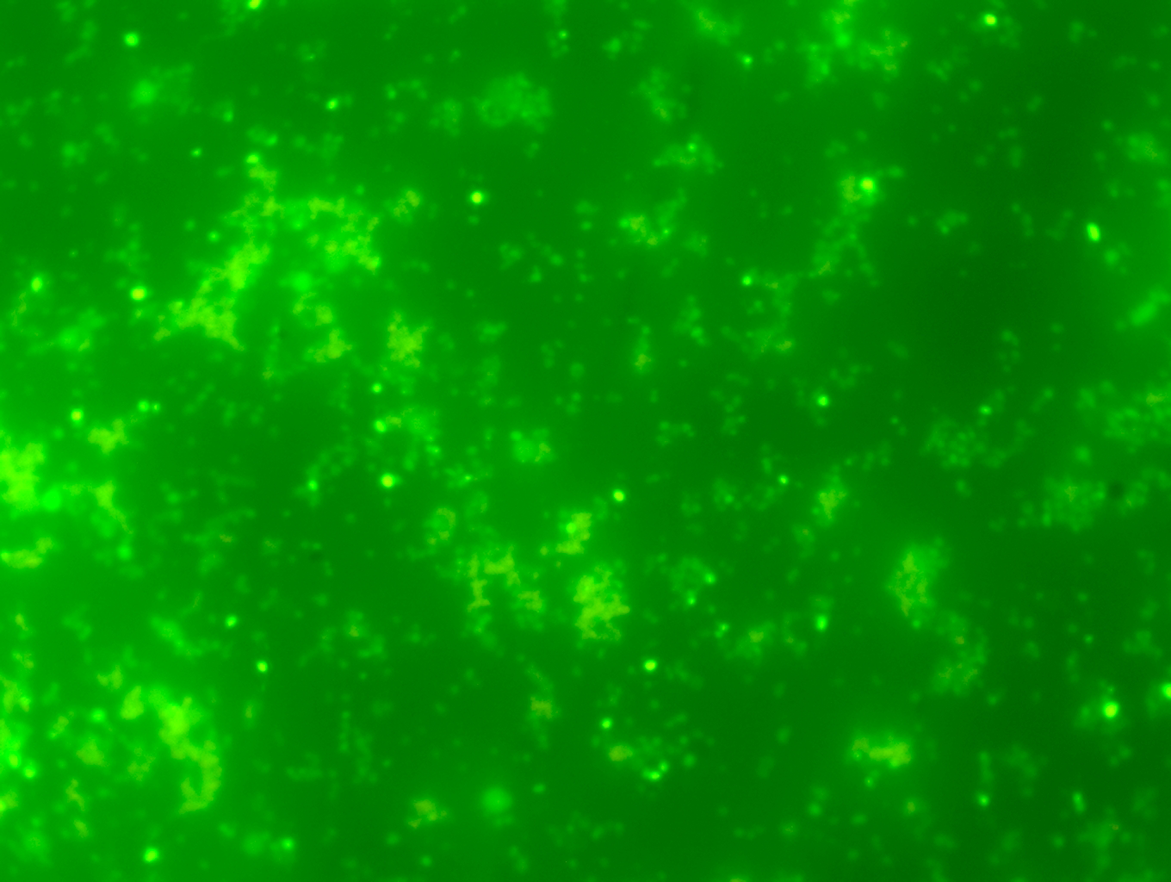

Supplement: Supplementary file 27 — Authors’ original file for figure 27 [file 40204_2012_15_MOESM27_ESM.tiff]

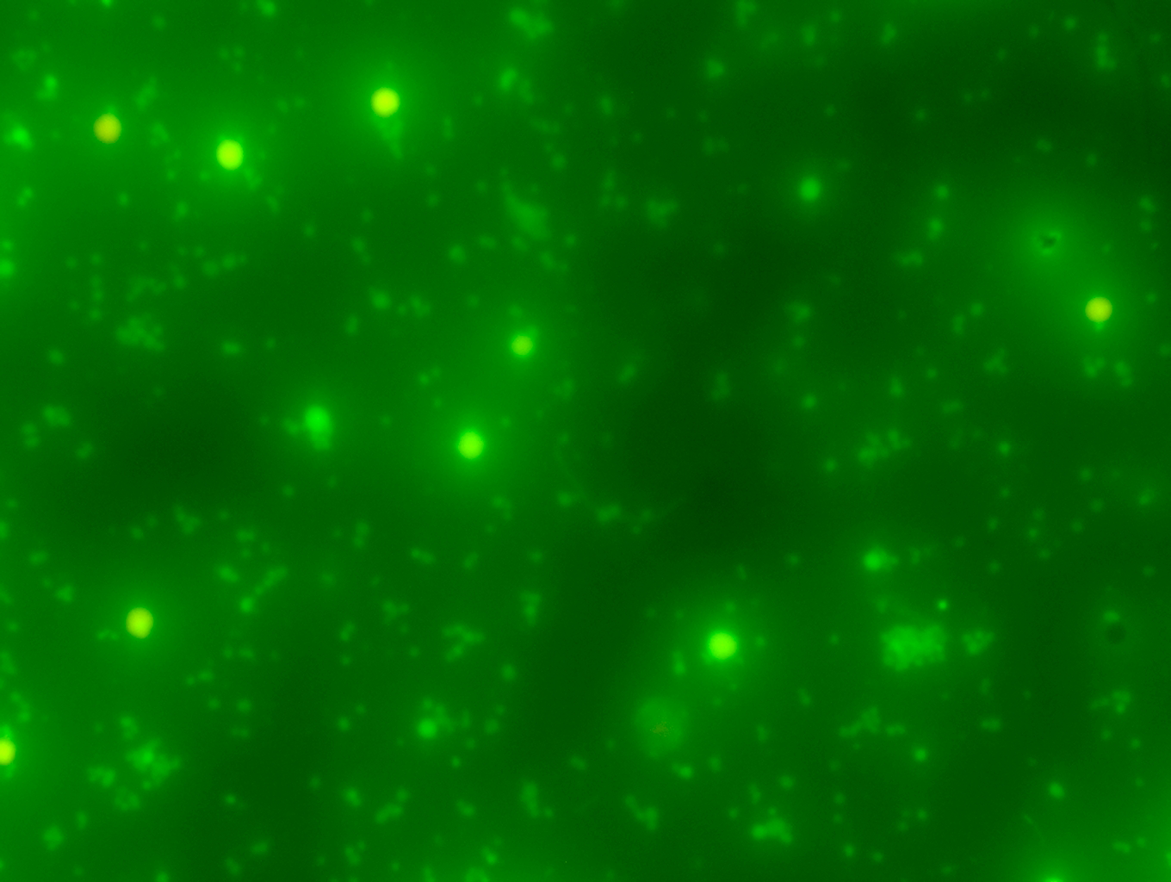

Supplement: Supplementary file 28 — Authors’ original file for figure 28 [file 40204_2012_15_MOESM28_ESM.tiff]

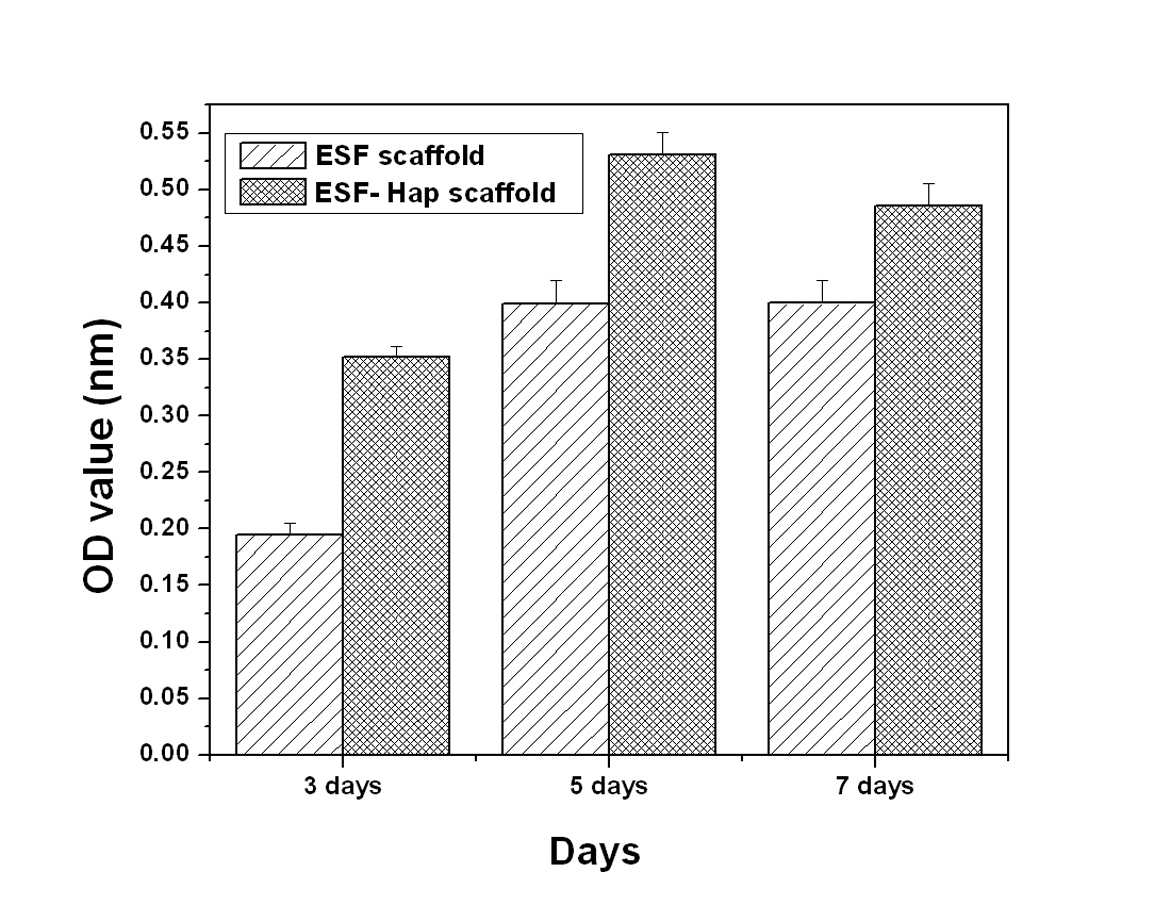

Supplement: Supplementary file 29 — Authors’ original file for figure 29 [file 40204_2012_15_MOESM29_ESM.tiff]
